# A rollover safety margin-based approach for quantifying the tractor-semitrailers’ emergency lane-changing response on expressway curves

**DOI:** 10.1371/journal.pone.0291783

**Published:** 2023-09-20

**Authors:** Wenzhen Lv, Jinliang Xu, Chao Gao

**Affiliations:** School of Highway, Chang’an University, Xi’an, Shannxi, China; Beijing Institute of Technology, CHINA

## Abstract

In emergency scenarios, lane changing can provide a considerable advantage over braking by aiding in the prevention of rear-end collisions. However, executing lane changes on horizontal curves might lead to rollover collisions. This study proposes a systematic methodology for quantifying the rollover safety margin during lane-changing maneuvers by encompassing the complex characteristics of vehicle-road interactions. Specifically, an enhanced six-degree-of-freedom vehicle dynamics model was developed for a tractor-semitrailer and integrates road superelevation. Using this model, the rollover safety margin reduction rate (*f*_S_) was calculated. The *f*_S_ represents the ratio of the difference between the lateral load transfer ratio margins under both reference state and emergency lane change conditions to the lateral load transfer ratio margin in the reference state. The reference state corresponds to vehicles maintaining 80 km·h^-1^ on a 270 m radius curve, while the emergency condition is defined as lane change durations of less than 4 seconds. The results reveal that emergency lane change maneuvers and roadway alignment significantly affect rollover safety margin. Shorter lane change duration, higher speed, and smaller radius worsen the rollover safety margin; these effects are further amplified when the lane change direction is opposite to the curve’s bending direction. When the tractor-semitrailer performs a lane change at 60 km·h^-1^ within a 4-second duration on a 600 m radius curve, the *f*_S_ exceeds 100%, indicating an imminent rollover. Consequently, this study contributes valuable evidence to the development of more reliable and secure lane-change strategies.

## 1. Introduction

The term "emergency lane-change maneuver" denotes a collision-avoidance technique initiated in response to an imminent collision, requiring a swift lane transition to evade the incident. In high-speed situations, this maneuver can be more effective than deceleration, as it demands less longitudinal displacement for collision avoidance [1]. Yuan et al.’s analysis supports this claim, suggesting that strategic lane-changes could potentially avert 24% of rear-end collisions [2]. Despite their benefits in averting rear-end collisions, executing safe emergency lane changes presents a significant challenge for tractor-semitrailers. This is due to their elevated center of mass and reduced maneuverability compared to other vehicle types, which increases their susceptibility to rollover collisions during lane changes. This should not be construed as advocating the abandonment of lane-change maneuvers for indiscriminate emergency braking under critical conditions. Rather, it underscores the imperative nature of investigating approaches and technologies targeted at preventing rollover incidents. Traditionally, to enhance the rollover stability of tractor-semitrailers, both active and passive anti-rollover technologies have been developed [3–5]. In recent times, the deployment of advanced driver-assistance systems incorporating automated lane-changing control strategies has emerged as an effective measure to circumvent the occurrence of undesirable driver behaviors when encountering emergency situations [6]. These strategies enable precise adjustment of a tractor-semitrailer’s lane change parameters, encompassing direction, speed, duration, and response time, based on the prevailing traffic conditions. However, such technologies mainly focus on the vehicle itself, neglecting the consequences of vehicle-road system interactions on rollover safety margin. From a systems theory perspective, vehicles and roads are essential components of complex transportation systems, each carrying distinct responsibilities and exerting different impacts on rollover safety [7]. It is evident that current research on lane-changing control strategies is not exhaustive; therefore, proposing a method to assess the impact of emergency lane-changing maneuvers for tractor-semitrailers on expressways holds significant importance. This approach more accurately captures the systemic properties of vehicle-road interactions, fostering a deeper comprehension of safety challenges during emergency lane changes. Moreover, this research significantly contributes to the development of reliable autonomous lane-change strategies, thereby broadening the spectrum of safety collision-avoidance tactics in emergency scenarios, and ensuring the safety and well-being of vehicle occupants.

Several studies have shown that there is a higher probability of rollover collisions on curves than on straight roads. Furthermore, the frequency of road accidents tends to increase as the radius of horizontal curves decreases [8, 9]. However, some studies have proposed that roads can still provide a sufficient rollover safety margin for vehicles even when the curve radius is at its minimum limit [10]. The main reason for this controversy is that the theoretical basis of [10] relies on the point-mass model for the quasi-static turning performance of rigid vehicles. This model makes several assumptions that do not accurately reflect reality, such as vehicles turning at a uniform speed along a circular trajectory. In fact, the emergency lane-changing maneuver triggered by unforeseen circumstances, including sudden braking by the front vehicle or objects being thrown out of the front vehicle’s window, can cause the vehicle to deviate from its circular trajectory. This deviation induces an additional lateral load transfer, increasing the risk of vehicle rollover. In recognition of this complexity, more advanced vehicle dynamics models have been introduced, such as the single-track and multi-body model [11, 12]. These innovative models aim to portray the vehicle’s instantaneous state and furnish a theoretical framework for appraising the rollover response characteristics under emergency lane-changing circumstances. Building on this complex dynamics model [13], investigated the lateral stability of a loaded vehicle and showed that rollover is highly sensitive to vehicle speed and steering angle. Several studies predicted the rollover phenomenon based on the turning radius [14, 15] and also proposed the maximum allowable speed for single trucks not to prevent rollover [16]. Collectively, these findings underscore that vehicle speed, curve radius, and instantaneous turning angle are integral variables in determining vehicle rollover stability. The aforementioned investigations have paved the way for further exploration and yielded crucial insights. However, the existing body of literature still lacks a comprehensive quantitative analysis that fully elucidates the extent to which these contributing factors influence rollover stability.

Variations in parameters, such as vehicle mass and center of gravity height, lead to significant variations in stability among diverse vehicle types. Tractor-semitrailers display inferior maneuverability and lower lateral stability in comparison to passenger cars due to high inertia, a high center of gravity, and rear amplification effects [17]. The challenges in enhancing the lateral stability of vehicles have prompted the development of innovative control strategies. Numerous studies have proposed active control strategies, namely active steering [5, 18, 19] and differential braking [3, 20], to enhance the lateral stability of the vehicle by imparting additional transverse moments. Furthermore, passive vehicle rollover protection systems relying on rollover warning indicators, such as time to collision [21] and load transfer ratio [22], have also been developed. A small number of studies have used B-spline curves [23] and cubic-degree polynomials [6] as lane change trajectories to develop lane change control strategies for autonomous trucks. These sophisticated vehicular systems have diminished the hazards concomitant with rollover incidents in tractor-semitrailers. Recent groundbreaking research includes the development of a comprehensive vehicle stability assessment system that covers longitudinal, yaw, and roll stability. By utilizing strong tracking unscented Kalman filters and conventional Kalman filters, this system can accurately estimate the longitudinal, lateral, and vertical tire forces, significantly contributing to vehicle safety under critical driving conditions [24]. A full X-by-wire chassis coordinated control scheme has also been introduced, improving ride comfort, handling performance, and rollover prevention ability [25]. Moreover, an event-triggered sideslip angle estimator has been designed using low-cost sensors, exhibiting enhanced estimation accuracy and reliability [26]. Despite these advancements, the geometric characteristics of horizontal curves remain an integral element affecting rollover crashes. Identifying the propensity for rollover within the complex interplay of the vehicle-road infrastructure remains a substantial challenge. This gap in the literature leads to the innovation of the present study: proposing a method for the real-time assessment of vehicle rollover safety margin that comprehensively considers the synergistic effects of vehicle-road collaboration. Unlike previous works, this approach emphasizes the dynamic relationship between vehicle parameters and road geometry, thus offering a more nuanced and accurate evaluation of rollover safety margin.

Conducted within an intricate research framework, this study innovatively proposes a method that embodies vehicle-road system attributes to quantify the extent to which emergency lane-changing strategies for tractor-semitrailers on horizontal curves influence rollover safety margins. This novel approach significantly enhances the existing understanding of this dynamic interaction and provides a comprehensive measure of safety impacts. In a methodological advancement, a multifaceted quantitative analysis is orchestrated. The core of this novel approach hinges on theoretical modeling, employing the rollover margin reduction rate as a nuanced metric to evaluate the effects of emergency lane-change maneuvers on the rollover safety margin. The analysis commences with the formulation of a superelevation modified transient single-track model, assimilating superelevation and front wheel turning angle as essential inputs, synergized with a lane change model in curves. These innovative models are remarkable in that they simultaneously integrate road geometric conditions and vehicle attributes. Such a fusion ensures an intricate and precise analysis of the rollover safety margin of the rollover safety margin—a reflection of systems engineering that elevates this study’s uniqueness. Following the establishment of these models, the rollover safety margin of tractor-semitrailers is meticulously calculated in the reference state, and the minimum rollover margin during emergency lane change in the curve is ascertained. By taking the differential between these two margins and normalizing it by the rollover margin in the reference state, the rollover margin reduction rate is computed. This inventive metric, going beyond conventional measures, stands as an intuitive, systemic indicator, encapsulating the impact of emergency lane-changing strategies on rollover safety margins for tractor-semitrailers in curves. The subsequent series of quantitative analyses illuminate the intricate relationship between emergency lane-change strategies for tractor-semitrailers on horizontal curves and the rollover safety margin. This exploration is further enriched by suggesting the potential applicability of the rollover safety margin reduction rate indicator in autonomous tractor-semitrailers. The inclusion of this innovative metric opens new vistas for future research and applications, reinforcing this study’s contribution to the scholarly dialogue in vehicle dynamics and road safety.

The remainder of the paper is structured as follows. Section 2 features the development of a single-track model with superelevation and a curve lane change model, and an index for quantifying the impact of emergency lane change strategies on rollover safety margin is introduced. In Section 3, the relationships between horizontal curve characteristics and emergency lane change strategies are explored in connection with rollover safety margin are examined. Section 4 provides the conclusion.

## 2. Materials and methods

This section begins by formulating a six-degree-of-freedom (6-DOF) dynamics model for the tractor-semitrailer, which incorporates a superelevation correction. Emergency lane change maneuvers are then defined, followed by the proposal of an index and methodology for assessing the rollover safety margin. Finally, a co-simulation is conducted using TruckSim and MATLAB/Simulink, thereby validating the proposed approach’s effectiveness in predicting real-world scenarios.

### 2.1 A 6-DOF dynamics model with superelevation variable

The objective of this study is to formulate a method that delineates the response characteristics of rollover safety margin for tractor-semitrailers during emergency lane-changing maneuvers on horizontal curves. Accomplishing this requires the development of an accurate and user-friendly dynamic model for tractor-semitrailers. Various models with different levels of complexity have been developed, including the point-mass model, the single-track model, and the multi-body model. The traditional point-mass model is limited to evaluating steady-state driving conditions and cannot assess transient states such as braking and lane changing. While the multi-body model can reflect the transient force state of each tire, it often suffers from unstable numerical solutions due to its high degree of freedom. On the other hand, the single-track model can accurately depict the transient response characteristics of lane change scenarios, while simultaneously offering high efficiency and stable numerical solutions. As a result, it is widely used in vehicle control and other research areas [11]. However, the single-track model has not yet taken into account the superelevation variable on curved roads. It is important to note that superelevation plays a crucial role in preventing rollover of loaded vehicles, as it induces a tilt in the opposite direction of lateral acceleration [27]. Thus, it is essential to consider the impact of superelevation on the lateral stability of the vehicle. Based on this rationale, we modify the single-track model by integrating the superelevation variable and develop an improved transient single-track model with front wheel turning angle and superelevation as input variables. This modified model is then utilized to assess the rollover safety margin of tractor-semitrailers under lane change scenarios.

In order to balance modeling accuracy with computational efficiency, several simplifications were incorporated into the development of the improved single-track model.

1. The body is simplified to two rigid bodies connected by traction pins, while the suspension is represented to a spring-damped structure, and the saddle is approximated to a linearly damped structure.
2. It is assumed that the steering and drive axles, as well as the saddle and trailer axle, are connected by rigid beams.
3. Symmetry is assumed for the forces about the longitudinal axis of the vehicle.
4. Front-wheel steering is assumed, with the steering characteristics modeled according to the Ackermann steering model.
5. The vehicle width is neglected, and each wheel of the steering axle, drive axle, and trailer axle is approximated by a centered wheel.
6. The slip angle of the tires is assumed to be within the range of ±0.2 rad, and the lateral acceleration of the vehicle is assumed to be less than 0.4 g.
7. Pitch caused by factors other than superelevation is ignored.
8. Air resistance and tire rolling resistance are neglected.
9. The front wheel turning angle and tire side angle are assumed to be small, such that their sine and tangent values can be approximated as themselves, and a cosine value of 1 is assumed.

**Fig 1:**
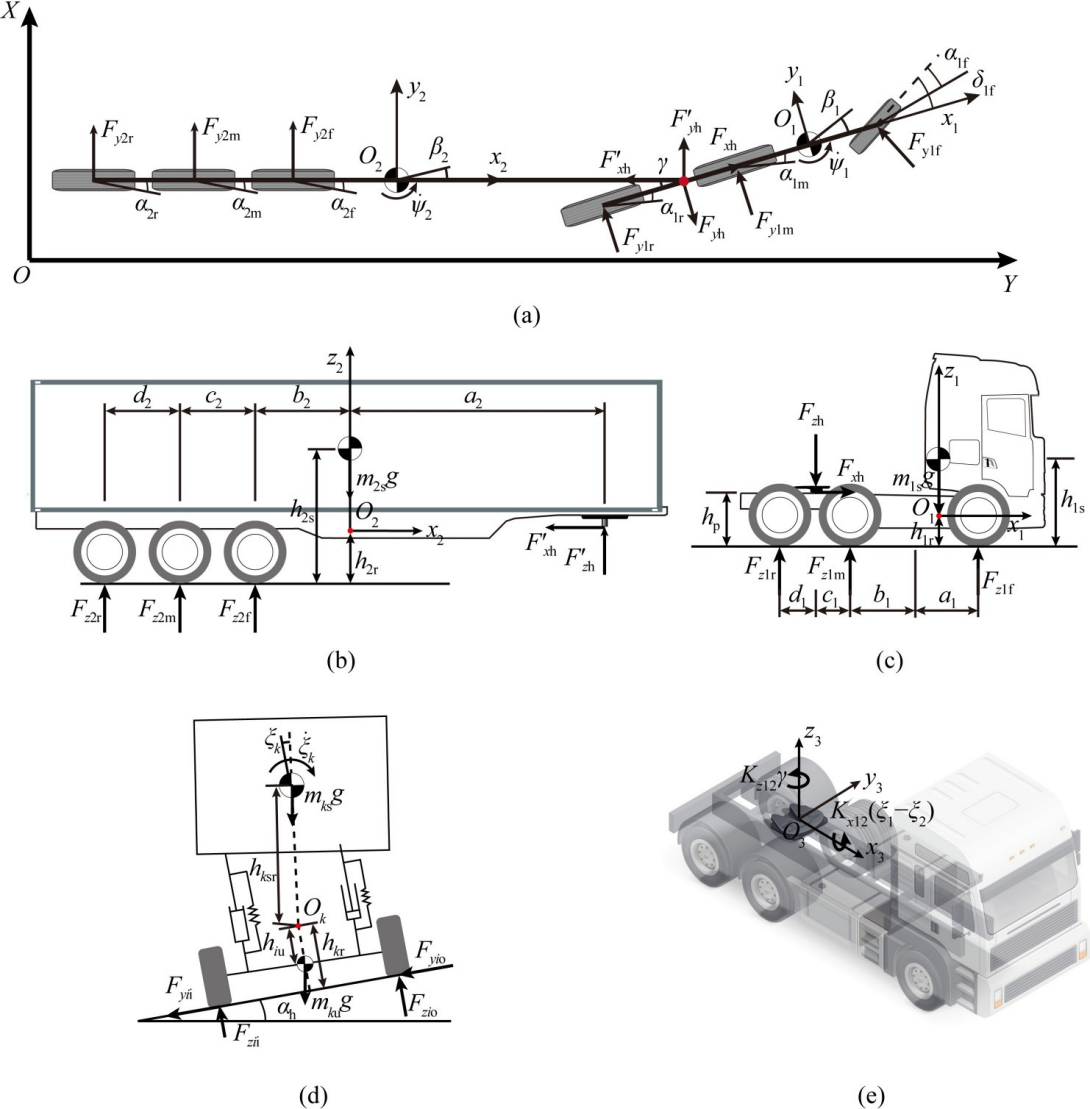
The 6-DOF model of the tractor-semitrailer. (a) Top view. (b) Side view of trailer. (c) Side view of tractor. (d) Rear view of tractor-semitrailer. (e) Saddle resistance moment.

The simplified single-track model, as shown in Fig 1, consists of 6-DOF, including the sideslip angle of the barycenter, the yaw angle, and the roll angle of the sprung mass for both the tractor and the trailer. To facilitate modeling analysis, the *XOY* Cartesian coordinate system was established, along with the local coordinate systems *x*_1_*o*_1_*y*_1_, *x*_2_*o*_2_*y*_2_, and *x*_3_*o*_3_*y*_3_. The International System of Units (SI) is utilized for all parameters in the mathematical equations involved in the modeling process, with angle variables represented in radians if not stated otherwise.

#### 1) Vehicle model

By utilizing the isolation method of modeling, the mechanical analysis of the tractor-semitrailer is illustrated in Fig 1. The lateral, yaw, and roll dynamics equations for both the tractor and the trailer can be derived by applying D’Alembert’s principle.

For the tractor

$$m_{1}v_{x1}\left({\dot{\beta }}_{1}+{\dot{\psi }}_{1}\right)-m_{s1}h_{1\text{sr}}{\ddot{\varphi }}_{1}=F_{y1\text{f}}+F_{y1\text{m}}+F_{y1\text{r}}-F_{y\text{h}}+m_{1}g\sin\left(\alpha_{\text{h}}\right)$$ (1)

$$I_{z1}{\ddot{\psi }}_{1}-I_{xz1}{\ddot{\xi }}_{1}=a_{1}F_{y1\text{f}}-b_{1}F_{y1\text{m}}-\left(b_{1}+c_{1}+d_{1}\right)F_{y1\text{r}}+F_{y\text{h}}\left(b_{1}+c_{1}\right)-K_{z\text{12}}\gamma$$ (2)

$$\begin{array}{l} \left(I_{1\text{s}xx}+m_{1\text{s}}h_{1\text{sr}}^{2}\right){\ddot{\xi }}_{1}-m_{1\text{s}}h_{1\text{sr}}\left(v_{x1}\left({\dot{\beta }}_{1}+{\dot{\psi }}_{1}\right)-h_{1\text{sr}}{\ddot{\xi }}_{1}-g\sin\left(\alpha_{\text{h}}\right)\right)-I_{1\text{s}xz}{\ddot{\psi }}_{1}\\ =-M_{1\xi }+m_{\text{1s}}h_{1\text{sr}}g\left(\xi_{1}-\alpha_{\text{h}}\right)+K_{x12}\left(\xi_{2}-\xi_{1}\right)+F_{y\text{h}}h_{1\text{cr}} \end{array}$$ (3)

and for the semitrailer

$$m_{2}v_{x2}\left({\dot{\beta }}_{2}+{\dot{\psi }}_{2}\right)-m_{2\text{s}}h_{2\text{sr}}{\ddot{\xi }}_{2}=F_{y2\text{f}}+F_{y2\text{m}}+F_{y2\text{r}}+F_{y\text{h}}^{\prime }\cos\left(\gamma \right)+F_{x\text{h}}^{\prime }\sin\left(\gamma \right)+m_{2}g\sin\left(\alpha_{\text{h}}\right)$$ (4)

$$I_{2zz}{\ddot{\psi }}_{2}-I_{2\text{s}xz}{\ddot{\varphi }}_{2}=-b_{2}F_{y2\text{f}}-\left(b_{2}+c_{2}\right)F_{y2\text{m}}-F_{y2\text{r}}\left(b_{2}+c_{2}+d_{2}\right)+F_{y\text{h}}^{\prime }a_{2}\cos\left(\gamma \right)+F_{x\text{h}}^{\prime }a_{2}\sin\left(\gamma \right)+K_{z\text{12}}\gamma$$ (5)

$$\begin{array}{l} \left(I_{2\text{s}xx}+m_{\text{2s}}h_{\text{2sr}}^{2}\right){\ddot{\xi }}_{2}-m_{2\text{s}}h_{2\text{sr}}\left(v_{x2}\left({\dot{\beta }}_{2}+{\dot{\psi }}_{2}\right)-h_{2\text{sr}}{\ddot{\xi }}_{2}-g\sin\left(\alpha_{\text{h}}\right)\right)-I_{2\text{s}xz}{\ddot{\psi }}_{2}\\ =-M_{\xi 2}+m_{2\text{s}}gh_{2\text{s}}\left(\xi_{2}-\alpha_{\text{h}}\right)-K_{x12}\left(\xi_{2}-\xi_{1}\right)-F_{y\text{h}}^{\prime }h_{2\text{cr}} \end{array}$$ (6)

Upon analyzing (1) and (4), it can be observed that the superelevation is beneficial in reducing the lateral acceleration of the vehicle when the lateral force on the vehicle’s weight acts in the opposite direction to the tire’s lateral force, and vice versa. Similarly, based on (3) and (6), the superelevation helps reduce the roll moment of the vehicle when the vehicle’s roll angle is opposite to the direction of the superelevation.

By approximating the tractor-semitrailer’s suspension system as a spring-damped system, the roll resistance moment induced by the suspension can be determined [11].


$$\left\{\begin{array}{l} M_{1\xi }=M_{\text{f}\xi }+M_{\text{r}\xi }=\left(K_{\text{f}}+K_{\text{r}}\right)\xi_{1}+\left(C_{\text{f}}+C_{\text{r}}\right){\dot{\xi }}_{1}=K_{\text{1}}\xi_{1}+C_{1}{\dot{\xi }}_{1}\\ M_{2\xi }=K_{\text{2}}\xi_{1}+C_{2}{\dot{\xi }}_{1} \end{array}\right.$$ (7)

The linear tire model for small sideslip angle is utilized to simulate the tire force, as detailed in [28]. The lateral force of each tire can be mathematically represented as follows:

$$\left\{\begin{array}{l} F_{y1\text{f}}=k_{1\text{f}}\alpha_{1\text{f}}=k_{1\text{f}}\left(\beta_{1}+\frac{a_{1}{\dot{\psi }}_{1}}{v_{x1}}-\delta_{1\text{f}}\right)\\ F_{y1\text{m}}=k_{1\text{m}}\alpha_{1\text{m}}=k_{1\text{m}}\left(\beta_{1}-\frac{b_{1}{\dot{\psi }}_{1}}{v_{x1}}\right)\\ F_{y1\text{r}}=k_{\text{1r}}\alpha_{\text{1r}}=k_{\text{1r}}\left(\beta_{1}-\frac{\left(b_{1}+c_{1}+d_{1}\right){\dot{\psi }}_{1}}{v_{x1}}\right)\\ F_{y2\text{f}}=k_{\text{2f}}\alpha_{2\text{f}}=k_{\text{2f}}\left(\beta_{2}-\frac{b_{2}{\dot{\psi }}_{2}}{v_{x2}}\right)\\ F_{y2\text{m}}=k_{\text{2m}}\alpha_{\text{2m}}=k_{2\text{m}}\left(\beta_{2}-\frac{\left(b_{2}+c_{2}\right){\dot{\psi }}_{2}}{v_{x2}}\right)\\ F_{y2\text{r}}=k_{\text{2r}}\alpha_{\text{2r}}=k_{\text{2r}}\left(\beta_{2}-\frac{\left(b_{2}+c_{2}+d_{2}\right){\dot{\psi }}_{2}}{v_{x2}}\right) \end{array}\right.$$ (8)

The longitudinal load transfer is not considered in this linear tire model, as the vehicle is assumed to undergo lane-change maneuvers at a constant speed.

A motion constraint between the tractor and the trailer exists, as outlined in [29]. This constraint can be expressed as follows:

$${\dot{\beta }}_{2}={\dot{\beta }}_{1}-\frac{h_{1\text{cr}}{\ddot{\xi }}_{1}}{v_{x1}}+\frac{h_{2\text{cr}}{\ddot{\xi }}_{2}}{v_{x2}}-\frac{\left(b_{1}+c_{1}\right){\ddot{\psi }}_{1}}{v_{x1}}-\frac{a_{2}{\ddot{\psi }}_{2}}{v_{x2}}+{\dot{\psi }}_{1}-{\dot{\psi }}_{2}$$ (9)

Assuming a small articulation angle, we consider *F*_*x*h_ = *F*′_*x*h_, *F*_*y*h_ = *F*′_*y*h_. In the analysis of the driving characteristics of the tractor-semitrailer and taking into account saddle parameters [30], concluded that *M*_*z*12_ has a negligible effect on the dynamic response of the vehicle and can be safely disregarded. Consequently, by substituting (8) into (1)-(6), the 6-DOF vehicle dynamics differential equations are obtained. The equations are then transformed into matrix form ***P***_**t**_***X***′_**t**_ + ***Q***_**t**_***X***_**t**_ = ***R***_**t**_***U***_**t**_ and ultimately expressed in state space.

$$\left\{\begin{array}{l} -P_{t}^{-1}Q_{t}X_{t}+P_{t}^{-1}R_{t}U_{t}=A_{t}X_{t}+B_{t}U_{t}\\ Y_{t}=C_{t}X_{t}+D_{t}U_{t} \end{array}\right.$$ (10)

where $Y_{t}=X_{t}={\left[\beta_{1},{\dot{\psi }}_{1},\xi_{1},{\dot{\xi }}_{1},\beta_{2},{\dot{\psi }}_{2},\xi_{2},{\dot{\xi }}_{2}\right]}^{\text{T}}$, $U_{t}={\left[\alpha_{\text{h}},\delta_{1\text{f}}\right]}^{\text{T}}$, and the coefficient matrices ***P*_t_**、***Q*_t_**、***R*_t_**、***C*_t_**, and ***D*_t_** are given in S1 File.

Eq (10) describes the dynamic response of the tractor-semitrailer when subjected to superelevation and front wheel-turn angle inputs. In previous research, the vehicle model that considered only the front wheel-turn angle input [11] was used to study the lateral stability of the vehicle and assess the accuracy of the 6-DOF dynamics model. For a comprehensive understanding of the vehicle model, consult the appended table detailing the symbols and their respective definitions. This table also includes the tire cornering stiffness and suspension roll stiffness determined through parameter identification methods.

#### 2) Trajectory model for lane-changing on horizontal curves

The lane-changing maneuvers for the tractor-semitrailer on curves consists of two distinct processes: the steady-state driving stage before the lane change and the actual lane-changing stage. To develop the trajectory model for lane-changing, it is essential to establish the relationship between the lane-changing trajectory and the front wheel angle in both stages.

In the steady-state driving stage, the tractor-semitrailer maintains its position in the lane at a constant front wheel angle and driving speed. The steering radius of the vehicle in this state is equivalent to the radius of the horizontal curve, and the trajectory of the vehicle is a circular path. The parameter for the radius of the circular trajectory is given by the following expression:

$$R\text{=}\frac{1}{k}=\left|\frac{{\left({\dot{x}}^{2}+{\dot{y}}^{2}\right)}^{\frac{3}{2}}}{\dot{x}\ddot{y}-\ddot{x}\dot{y}}\right|$$ (11)

with,

$$\left\{\begin{array}{l} \dot{x}=v_{x}{}_{1}\cos\left(\psi_{1}\right)-v_{x}{}_{1}\tan\left(\beta_{1}\right)\sin\left(\psi_{1}\right)\\ \dot{y}=v_{x}{}_{1}\sin\left(\psi_{1}\right)+v_{x}{}_{1}\tan\left(\beta_{1}\right)\cos\left(\psi_{1}\right) \end{array}\right.$$ (12)

where the parameters *δ*_c_, *R*, and *k* correspond to the front wheel turning angle, curve radius, and curvature of the vehicle during steady-state turning. The variables *x* and *y* represent the instantaneous positions of the vehicle in the Cartesian coordinate system.

**Fig 2:**
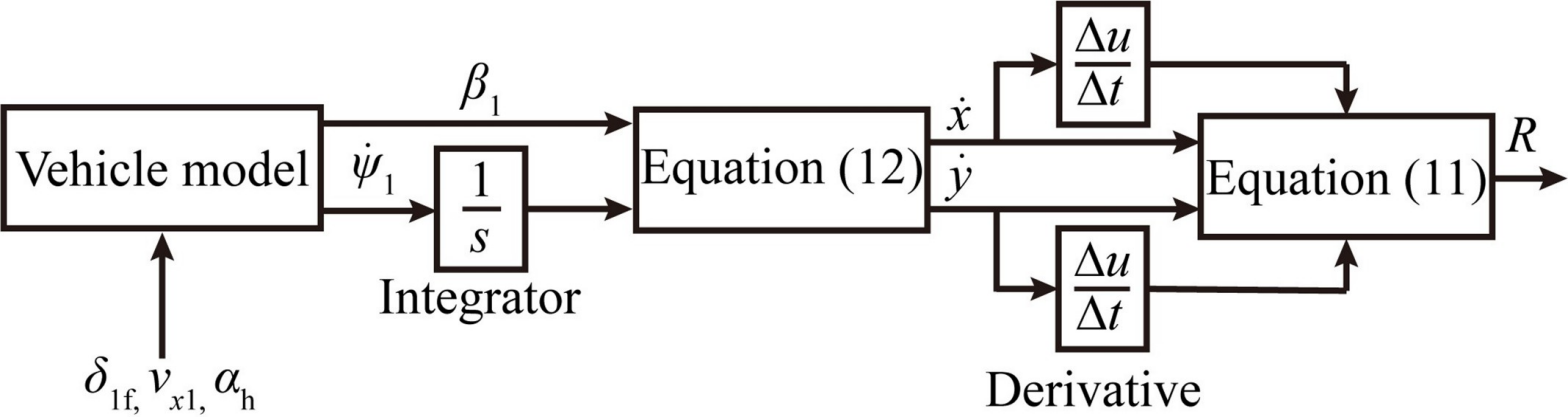
3D look-up table production process.

Next, we establish the relationship between the curve radius and the front wheel angle by making a 3D look-up table. As shown in Fig 2, we enter a multi-array array [*δ*_1f_, *v*_*x*1_, *α*_h_] to obtain the front wheel turning angle lookup table corresponding to the curve radius.

For the lane-changing stage, the front wheel angle shifts from the steady state to the transient state, resulting in an instantaneous change in the turning radius. Therefore, the traditional method of determining the front wheel angle based on the turning radius is no longer applicable. Instead, the approach based on the lane-changing trajectory provides a solution for capturing the instantaneous response characteristics of the front wheel angle. Several parametric equations have been proposed to describe the lane-changing trajectory of vehicles; among them, the B-spline curve exhibits the fundamental characteristics of vehicle lane-changing [23]. For this study, we employ the cubic B-spline curve as the trajectory equation for the lane change.

$$f(t)=\left\{\begin{array}{l} 0,t<t_{0}\\ e_{0}+e_{1}t+e_{2}t^{2}+e_{3}t^{3},t\in [t_{0},t_{1})\\ 3.75,t\geq t_{1} \end{array}\right.$$ (13)

where *f*(*t*) represents a cubic spline function, *t* denotes the simulation time, *t*_0_ denotes the start time of the lane change, *t*_1_ denotes the end time of the lane change, and *e*_0_, *e*_1_, *e*_2_ and *e*_3_ denote the cubic spline parameters.

**Fig 3:**
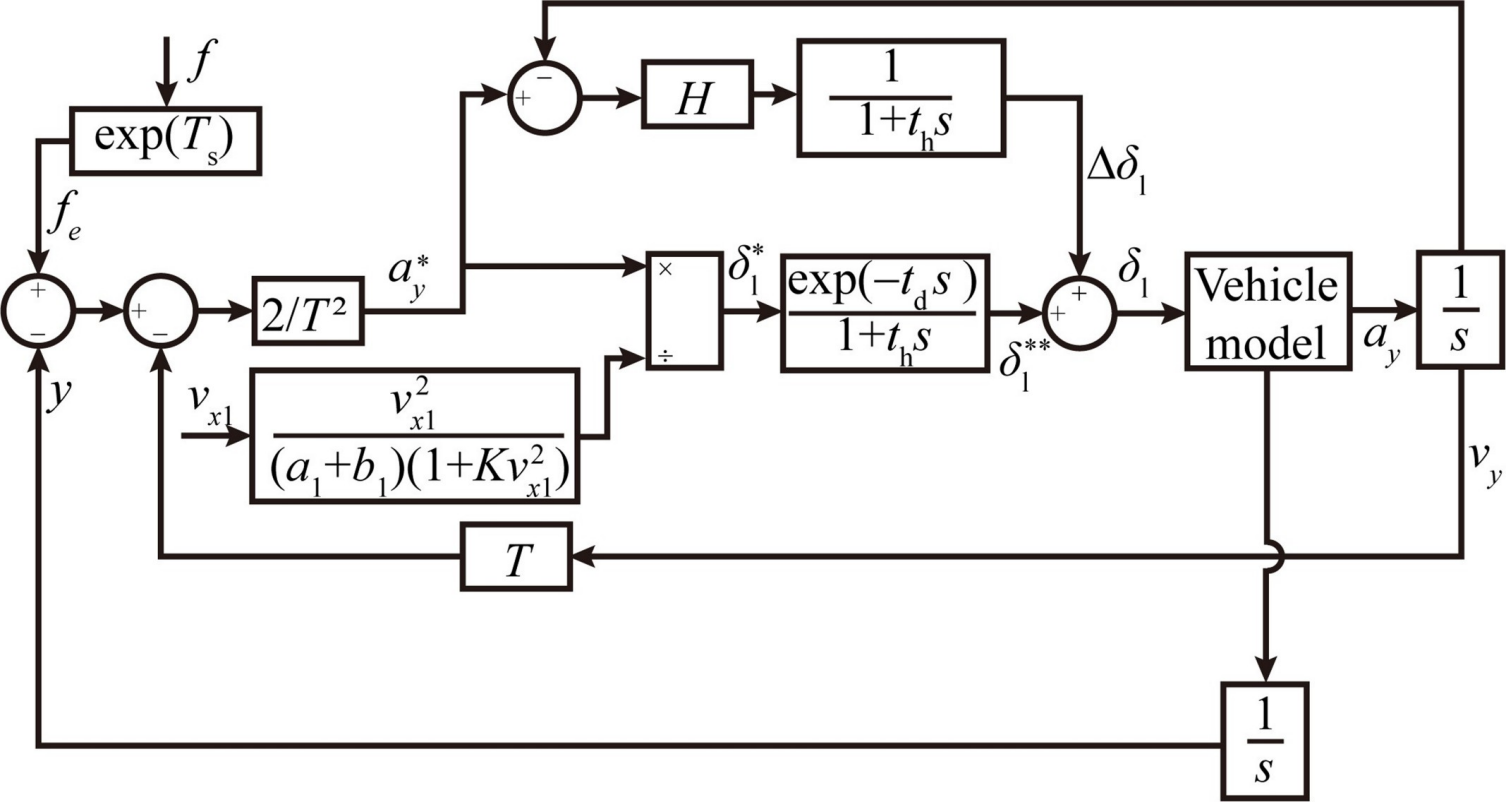
Model for tracking and controlling lane change trajectories.

It is important to note that the single-point preview control algorithm can be a useful tool in modeling the lane-changing maneuvers of vehicles, as it accounts for various factors that can influence the driver’s decision-making process (Fig 3). These factors include the pre-sighting time, the perception time, and the execution time. By optimizing the front wheel turning angle based on the desired trajectory and the current state of the vehicle, the algorithm can help to ensure that the vehicle follows the desired trajectory as closely as possible while also taking into account the driver’s response characteristics. Thus, the algorithm is a valuable tool for simulating the maneuverability of vehicles in lane-changing scenarios. More information about the single-point preview control algorithm can be found in [31].

$$\delta_{\text{l}}=\left(\left(\frac{2}{T^{2}}[f(t+T)-y(t)-T\dot{y}(t)]\right)\frac{1}{G_{\text{a}y}}\right)\frac{\exp\left(-t_{\text{d}}s\right)}{1+t_{\text{h}}s}$$ (14)

with,

$$G_{ay}=\frac{v_{x1}^{2}}{\left(a_{1}+b_{1}\right)\left(1+Kv_{x1}^{2}\right)}$$ (15)

where *δ*_*l*_ represents the front wheel turning angle during lane change, *G*_a*y*_ is the steady-state gain of the front wheel turning angle, *T* is the pre-sighting time, *y*(*t*) and $\dot{y}$(*t*) represent the lateral position and lateral velocity of the vehicle at time *t*, respectively. The sensing time, typically 0.2–0.6 s, is denoted as *t*_d_, while *t*_h_ represents the execution time, typically 0.05–0.20 s. exp(-*t*_d_*s*) and 1/(1+*t*_h_*s*) represent the transfer functions. Finally, *K* is the stability coefficient, which ranges from 0.002 to 0.004.

**Fig 4:**
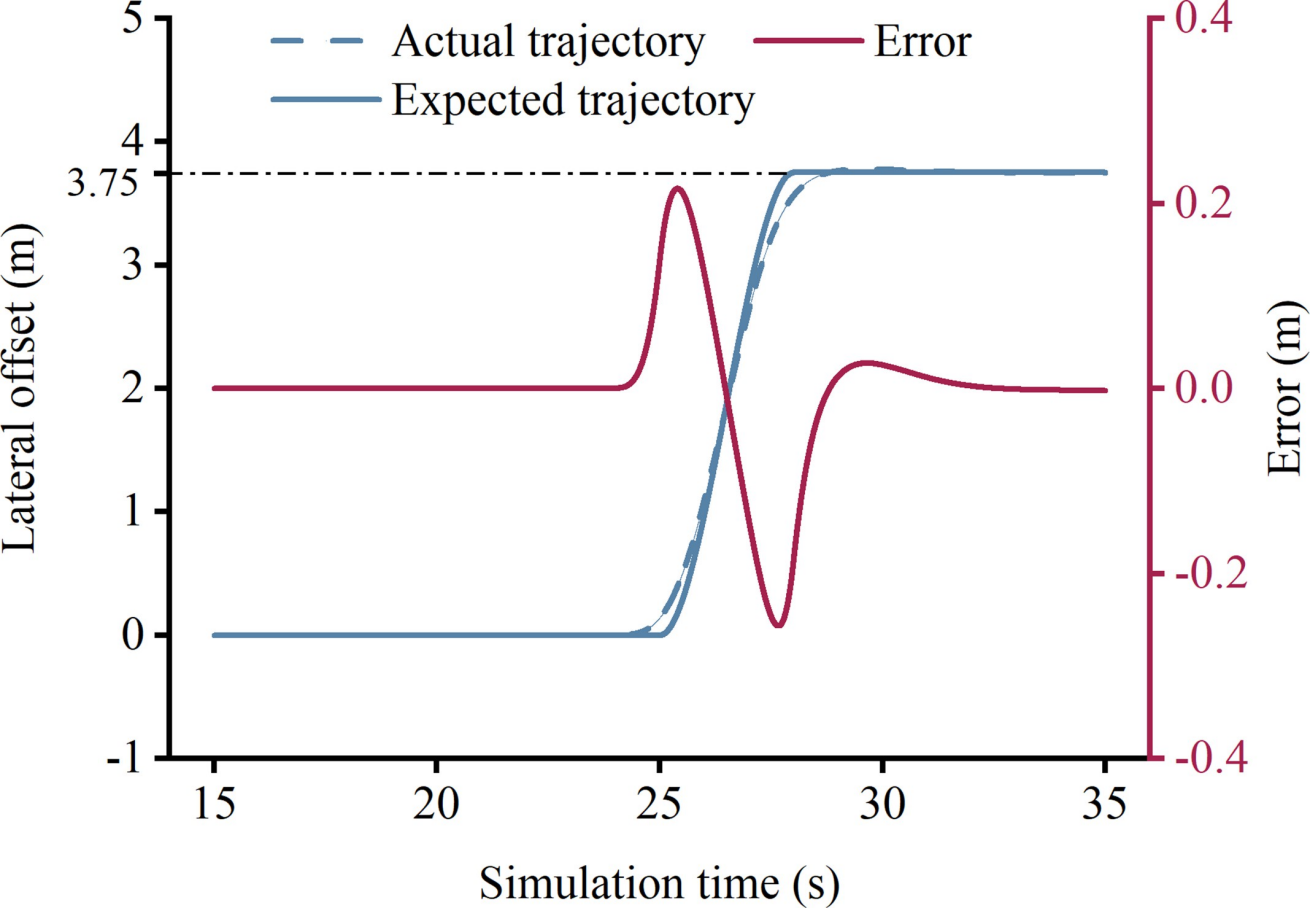
Expected and actual trajectories of a lane change.

Fig 4 showcases the tracking control effect of a lane change trajectory for the tractor-semitrailer, with a maximum deviation of 0.23 m. This level of error is deemed acceptable, given that the 3.75 m width of a highway lane encompasses the vehicle’s width and lateral clearance. Notably, tractor-semitrailers have a width limit of 2.5 m, which means that the lateral clearance offered by the lane exceeds 1 m. Furthermore, Fig 4 attests to the applicability of the path tracking control algorithm, initially developed for intelligent passenger cars, to tractor-semitrailers.

The scope of our study concerns emergency lane-changing strategies in horizontal curves, which necessitates accounting for the track offset induced by the curvature when determining the front wheel angle necessary for a lane change. As a result, the front wheel angle of the tractor-semitrailer during a lane change can be expressed as follows:

$$\delta_{1\text{f}}\text{=}\delta_{\text{c}}\pm \delta_{\text{l}}$$ (16)

The algebraic signs in the equation correspond to the direction of the lane change. Specifically, the positive sign signifies a lane change that follows the curvature, while the negative sign indicates a lane change in the opposite direction. Further elucidation on the nuanced aspects of the lane change direction will be provided in the subsequent section.

### 2.2 Safety margin evaluation method for rollover under emergency lane change maneuvers

#### 2.2.1 Characterization parameters for emergency lane-changing maneuvers

The aim of this study is to investigate the extent to which emergency lane-changing strategies affect the rollover safety margin. An emergency lane change is defined as the immediate steering maneuver executed by a vehicle to avoid collisions when confronted with unforeseen events, such as fallen objects appearing in the field of vision or abrupt braking by a leading vehicle. In vehicle stability studies, parameters such as the maximum instantaneous steering angle of the front wheel [32], steering frequency [33], and lane change duration (*LCD*) [34] have been used to gauge lane-change urgency. Although the maximum instantaneous steering angle provides information about the turning angle during a lane change, it fails as an accurate urgency indicator since the same angle may imply different urgency levels depending on factors like speed and road conditions. Similarly, steering frequency falls short as an urgency indicator, as the vehicle’s force and stability can widely vary with factors such as speed and turning radius. In contrast, the *LCD* offers a direct measure of lane-change urgency: the shorter the *LCD*, the more substantial the lateral impact, and the more marked the effect on vehicle stability. Therefore, this study adopts the *LCD* as the characteristic parameter for evaluating emergency lane change maneuvers. Data on *LCD*, especially in emergency situations, is scarce. Previous studies exploring *LCD* through field tests and simulations suggest that typical *LCD* is 5–6 seconds [35–37], while Toledo [38] found the shortest *LCD* for trucks to be 1.6 seconds. Although these studies do not explicitly assess urgency, it can be deduced that emergency *LCD* might be less than 5–6 seconds. Time to Collision (TTC), representing the remaining time before a possible collision with a leading vehicle in the same lane under current speed and path conditions, typically falls within a 3–5 second range for most drivers [39]. This implies that the *LCD* in emergency situations may be less than 5 seconds (considering visual search and decision time), leading this study to regard the *LCD* under 4 seconds as indicative of emergency maneuvers. Three emergency lane-changing maneuvers are selected for analysis, with *LCD*s of 2, 3, and 4 seconds, respectively. Given that some studies have determined that driving speed remains constant during lane-changing in field tests [40], variable-speed lane-changing scenarios are not considered in this investigation.

**Fig 5:**
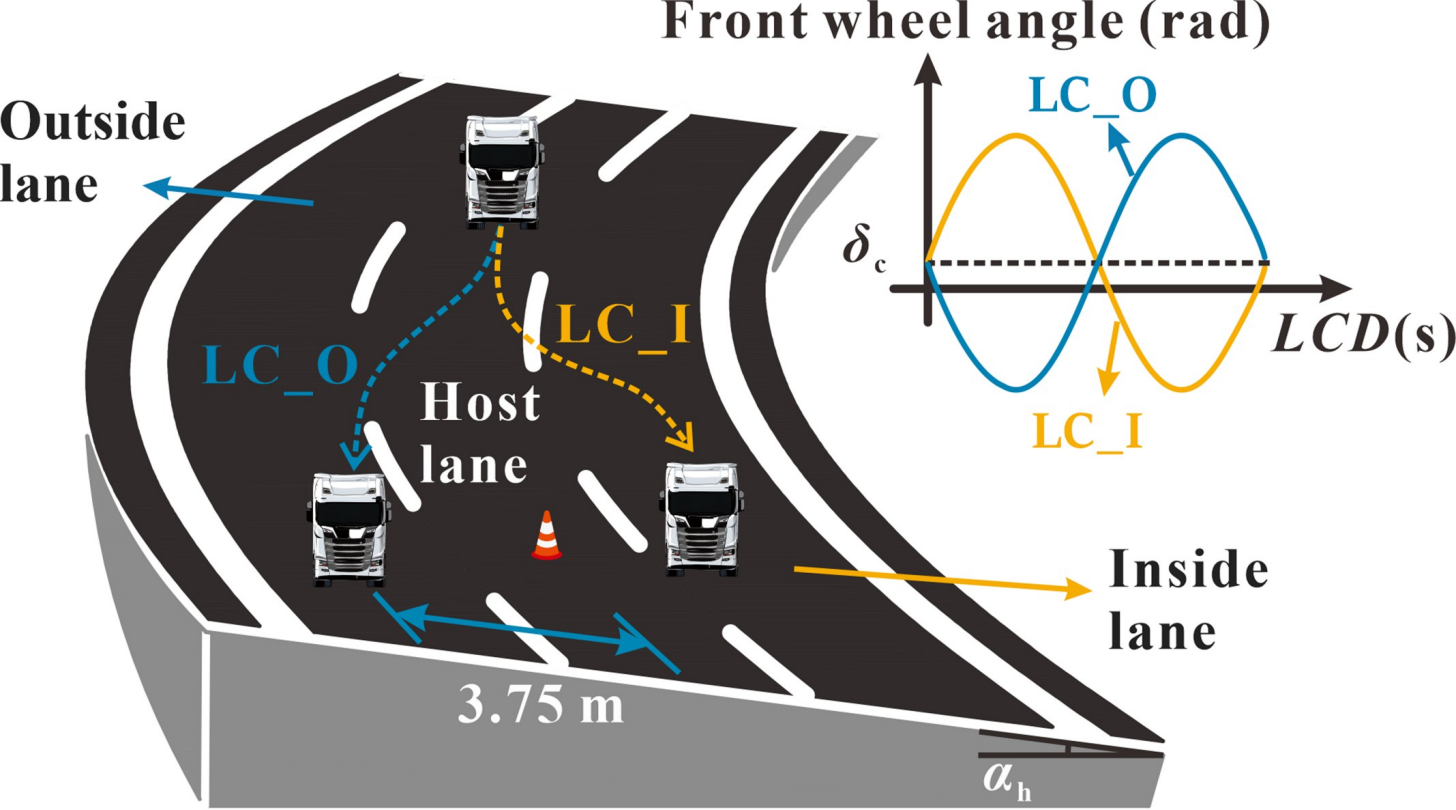
Two directions for lane change maneuvers.

An important factor to consider in rollover safety during lane-changing maneuvers is that superelevation can have both positive and negative effects, as illustrated in Fig 5. If the steering wheel turns in the same direction as the curve bend, superelevation serves as a stabilizing factor, reducing the vehicle’s propensity for rollover. Conversely, should the steering wheel turn in the direction opposite to that of the curve bend, superelevation manifests as a destabilizing factor, increasing the vehicle’s rollover tendency. The integration of the superelevation variable into the classic single-track model is both necessary and beneficial to enhance the accuracy of assessing the rollover safety margin. Consequently, this study investigates two lane change directions: LC_O, representing when the steering wheel turns in the direction opposite to the curve bend, and LC_I, denoting when the steering wheel turns in the same direction as the curve bend.

#### 2.2.2 Evaluation criteria and methodology for rollover safety margin

Systems theory suggests that rollover collisions are influenced by vehicular factors and road conditions at the event location [7]. Despite this, current research on lane-changing control strategies predominantly focuses on objective functions and predefined constraints associated with vehicle transient characteristics [41, 42], thereby neglecting the influence of road conditions on rollover safety. Lamm et al. [43] proposed a dynamic consistency index for reflecting safety during driving, employing a safety margin (representing the difference between the supply and demand values of the lateral friction coefficient) to represent the vehicle-road system’s dynamic safety level. However, this index, designed for vehicle skidding, cannot be directly applied to rollover safety margin research, and its quantification method falls short in capturing the effect of emergency lane-changing maneuvers and road alignment conditions on rollover safety margin. Consequently, assessing the impact of various emergency lane-changing strategies and road alignment conditions on rollover safety margin remains an intricate challenge. In highway geometric design, the minimum radius limit for a circular curve is predicated on the assumption that vehicles, at design speed with a constant turning radius, can avoid sideslip or rollover incidents [44]. Some studies also suggest that the minimum limit radius for highways provides sufficient rollover safety margin for vehicles operating under design conditions [10]. Hence, this study introduces the rollover safety margin reduction rate (*f*_S_), drawing on previously mentioned geometric features and driving conditions, to measure the effect of emergency lane change strategies for tractor-semitrailers on horizontal curves. This metric, delineated in (17), expresses the ratio of the difference between the rollover margins during a lane-change maneuver and the reference state to the reference state’s rollover margin. When *f*_S_ > 1, it indicates that the emergency lane change strategy may lead to a vehicle rollover. On the other hand, when 0 < *f*_S_ < 1, it suggests that the vehicle will not rollover under the emergency lane change strategy. If *f*_S_ = 0, it implies that the rollover safety margin under the emergency lane change strategy remains in the reference state.


$$f_{\text{S}}=\frac{\left(f_{\text{T}}-f_{\text{B}}\right)-\left(f_{\text{T}}-f\right)}{f_{\text{T}}-f_{\text{B}}}\times 100\text{\%}$$ (17)

Eq (17) represents a function that relates the rollover threshold (*f*_T_), the rollover basic value (*f*_B_), and the actual rollover state value (*f*). To precisely depict the rollover state of the tractor-semitrailer, this study utilizes the lateral load transfer ratio (*LTR*) [45], representing the vehicle’s instantaneous characteristics. The *LTR* is defined as the ratio between the difference and the sum of the normal load of the inner and outer tires. Eq (18) presents the mathematical expression of *LTR*. When *LTR* = ±1, the tires on one side of the vehicle lift, and the vehicle approaches the critical rollover state.


$$LTR=\frac{F_{z\text{o}}-F_{z\text{i}}}{F_{z\text{o}}+F_{z\text{i}}}$$ (18)

It can be observed from (17)-(18) that the accuracy of the rollover safety assessment result is determined by the precision of *f* and *f*_T_. The 6-DOF dynamic model serves as an effective means for obtaining a more precise *f*, as it can track the transient state of the vehicle and reflect the force characteristics of each axle, especially the trailer axle, which cannot be captured by the conventional point-mass model.

As defined by (18), the *LTR* value represents the lateral load transfer of a single axle. However, during a lane change, the *LTR* value of any axle of the tractor-semitrailer may reach ±1, a condition that could lead to the vehicle overturning due to unforeseen disturbances. Existing studies have shown that the maximum *LTR* value is not reached simultaneously across all axles [46]. Therefore, (19) is employed to calculate the maximum value of the absolute *LTR* for each axle during a lane change. The value of *f*_B_ is considered as the *f* value generated when the vehicle drives along the minimum circular curve radius at the designed speed.


$$f=\underset{i}{\max}\left(\underset{j}{\max}\left|LTR_{j}\right|\right)\;i=\text{f,r,t}\;j=1,2,3\cdot \cdot \cdot$$ (19)

The determination of the *f*_T_ presents a significant challenge, with implications for safety margin assessment. Although an *LTR* value of ±1 signifies the critical state of vehicle rollover, scholars typically examine *LTR* values ranging from 0.8 to 0.9 when investigating active safety control strategies for vehicle lateral stability [47, 48]. This approach is taken because *LTR* offers greater precision in the linear region and becomes more challenging to control as it approaches the critical rollover state. In this study, *f*_T_ is considered to be 0.85. As (17) represents a monotonically increasing function, variations in the threshold size do not influence the trend in rollover safety. A refined estimation of *f*_T_ will be the subject of future research.

It is essential to recognize that *LTR* cannot be directly obtained from the aforementioned 6-DOF model. This paper proceeds to derive a calculation method for *LTR*. Although methods to estimate *LTR* have been proposed in [49, 50], the approach presented in [49] does not account for roll dynamics and therefore fails to capture the instantaneous roll state of the tractor-semitrailer during a lane change. On the other hand, the equation proposed in [50] to estimate *LTR*, *LTR* = 2(*Kξ* + *C*$\dot{\xi }$)/*mgB*, where *B* is the wheelbase, *ξ* and $\dot{\xi }$ are the roll angle and roll rate of the sprung mass, and *K* and *C* are the roll angle stiffness and roll angle damping of the suspension, respectively, still does not account for lateral dynamics in predicting rollover. In [29], both lateral and roll dynamics are considered, and lateral acceleration is used as a key factor to predict rollover. Despite these advancements, prior research has consistently overlooked specific lateral features of horizontal curves, namely superelevation, which is an engineering measure designed to partially counteract the lateral forces on vehicles in steady state travel on horizontal curves. This oversight has led to an underestimation of lateral stability in vehicles on curves. Subsequent sections will introduce a novel *LTR* that integrates roadway superelevation as a variable, all the while considering the dynamic characteristics of the vehicle.

The moment balance equations can be derived for the unsprung masses associated with the tractor’s steering axle, drive axle, and trailer axle, based on the force exerted on the unsprung mass.


$$\Delta F_{z\text{f}}=\frac{F_{z\text{fo}}-F_{z\text{fi}}}{2}=\frac{m_{\text{fu}}h_{\text{fu}}\left(v_{x1}\left({\dot{\beta }}_{1}+{\dot{\psi }}_{1}\right)-g\sin\left(\alpha_{\text{h}}\right)\right)+F_{1\text{f}}h_{\text{1r}}+M_{\text{f}\xi }}{B_{\text{f}}}$$ (20)

$$\Delta F_{z\text{r}}=\frac{F_{z\text{ro}}-F_{z\text{ri}}}{2}=\frac{m_{\text{ru}}h_{\text{ru}}\left(v_{x1}\left({\dot{\beta }}_{1}+{\dot{\psi }}_{1}\right)-g\sin\left(\alpha_{\text{h}}\right)\right)+\left(F_{1\text{m}}+F_{1\text{r}}\right)h_{\text{1r}}+M_{\text{r}\xi }}{B_{\text{r}}}$$ (21)

$$\Delta F_{z\text{t}}=\frac{F_{z\text{to}}-F_{z\text{ti}}}{2}=\frac{m_{\text{tu}}h_{\text{tu}}\left(v_{x2}\left({\dot{\beta }}_{2}+{\dot{\psi }}_{2}\right)-g\sin\left(\alpha_{\text{h}}\right)\right)+\left(F_{2\text{f}}+F_{2\text{m}}+F_{2\text{r}}\right)h_{\text{2r}}+M_{\text{2}\xi }}{B_{\text{t}}}$$ (22)

The force transfer between the tractor and trailer takes place through the saddle, and from this relationship, the normal load exerted on each axle of the tractor-semitrailer can be defined as follows:

$$F_{z\text{f}}=\frac{m_{1}g\left(b_{1}+c_{1}+d_{1}\right)}{a_{1}+b_{1}+c_{1}+d_{1}}+\frac{m_{2}g\left(b_{2}+c_{2}+d_{2}\right)}{a_{2}+b_{2}+c_{2}+d_{2}}\frac{d_{1}}{a_{1}+b_{1}+c_{1}+d_{1}}$$ (23)

$$F_{z\text{r}}=\frac{m_{1}ga_{1}}{a_{1}+b_{1}+c_{1}+d_{1}}+\frac{m_{2}g\left(b_{2}+c_{2}+d_{2}\right)}{a_{2}+b_{2}+c_{2}+d_{2}}\frac{a_{1}+b_{1}+c_{1}}{a_{1}+b_{1}+c_{1}+d_{1}}$$ (24)

$$F_{z\text{t}}=\frac{m_{2}ga_{2}}{a_{2}+b_{2}+c_{2}+d_{2}}$$ (25)

The *LTR* of the tractor-semitrailer during a lane change can be determined by solving (18) and (20)-(25).


$$LTR_{i}=\frac{2\Delta F_{zi}}{F_{zi}}\;i=\text{f,r,t}$$ (26)

**Fig 6:**
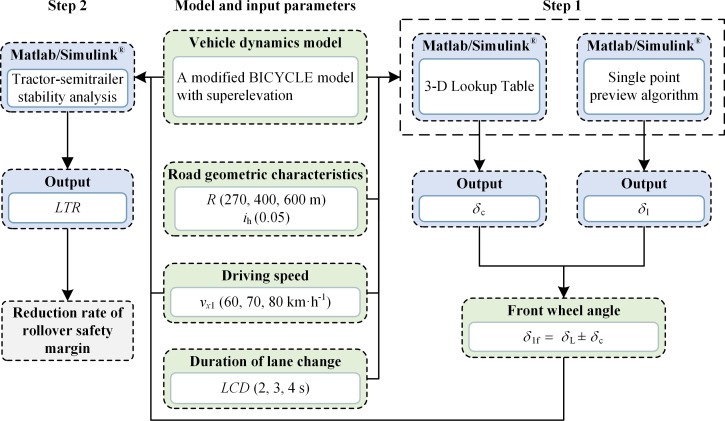
Calculation process of reduction rate of rollover safety margin.

The value of *f*_S_, obtained by solving the differential equations of vehicle dynamics, is generally complex to calculate analytically. However, numerical methods such as Runge-Kutta offer a reliable framework for calculating *f*_S_. MATLAB/Simulink^®^ provides a suite of standard numerical techniques and modular diagrams that augment modeling efficiency and are extensively applied in fields such as vehicle stability analysis and autonomous driving [47]. Fig 6 illustrates the utilization of MATLAB/Simulink^®^ to compute the *f*_S_ value. The 6-DOF vehicle model employs tractor-semitrailer parameters derived from [11], as detailed in S1 File. The road model should authentically represent the actual scenario, and as per the Technical Standard of Highway Engineering (JTG B01-2014), the minimum limit radius at a design speed of 80 km·h^-1^ is 270 m. In this study, the curve radii in the road model are set to 270 m, 400 m, and 600 m, with a maximum superelevation of 5%. The corresponding speeds are designated as 60 km·h^-1^, 70 km·h^-1^, and 80 km·h^-1^, and are converted to international units for the simulation.

### 2.3 Model validation through co-simulation between TruckSim^®^ and MATLAB/Simulink^®^

**Fig 7:**
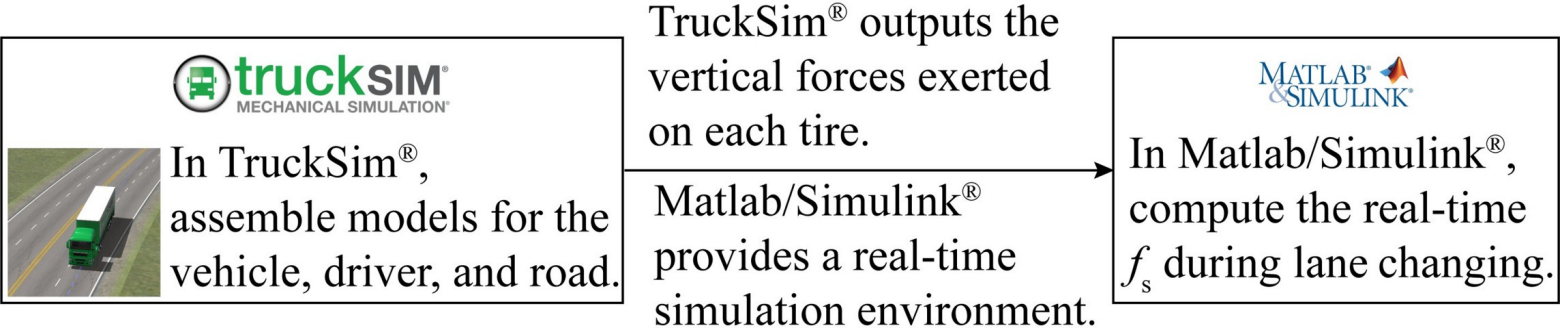
The co-simulation platform between TruckSim^®^ and MATLAB/Simulink^®^.

To ascertain the accuracy of the rollover safety margin assessment method based on the enhanced 6-DOF model, simulation experiments are conducted in varying lane-change directions using TruckSim^®^ and MATLAB/Simulink^®^. The verification process for the co-simulation is outlined as follows: The nonlinear tractor-semitrailer model is formulated using TruckSim^®^, software developed by the Mechanical Simulation Company. Known for precision validation, it boasts a high-fidelity nonlinear vehicle dynamics model validated through diverse experimental tests and is extensively employed in semitrailer stability research [51]. The selected vehicle model, comprising a 3A Cab Over and a 3A Euro Trailer, includes the tractor’s steering axle, drive axle, and semi-trailer axle, with parameters detailed in the S1 File. The driver model, configured to maintain a constant speed of 80 km·h^-1^ without braking maneuvers. The driver’s path-following model encompasses scenarios for driving along the lane (used for calibrating *f*_B_) and executing a lane-changing maneuver, with the lane change distance is 66.67 m, corresponding to a *LCD* of 3 s. The road model, designed as a dual-direction, four-lane road, includes a superelevation of 8% and circular curve radii of 270 meters (for calibrating *f*_B_) and 600 m. Finally, through the interface between TruckSim^®^ and MATLAB/Simulink^®^, the rollover safety margin assessment model is connected with the vehicle model and simulation environment in TruckSim^®^ via MATLAB/Simulink^®^, constituting a simulation platform for conducting joint simulation verification experiments, as shown in Fig 7.

**Fig 8:**
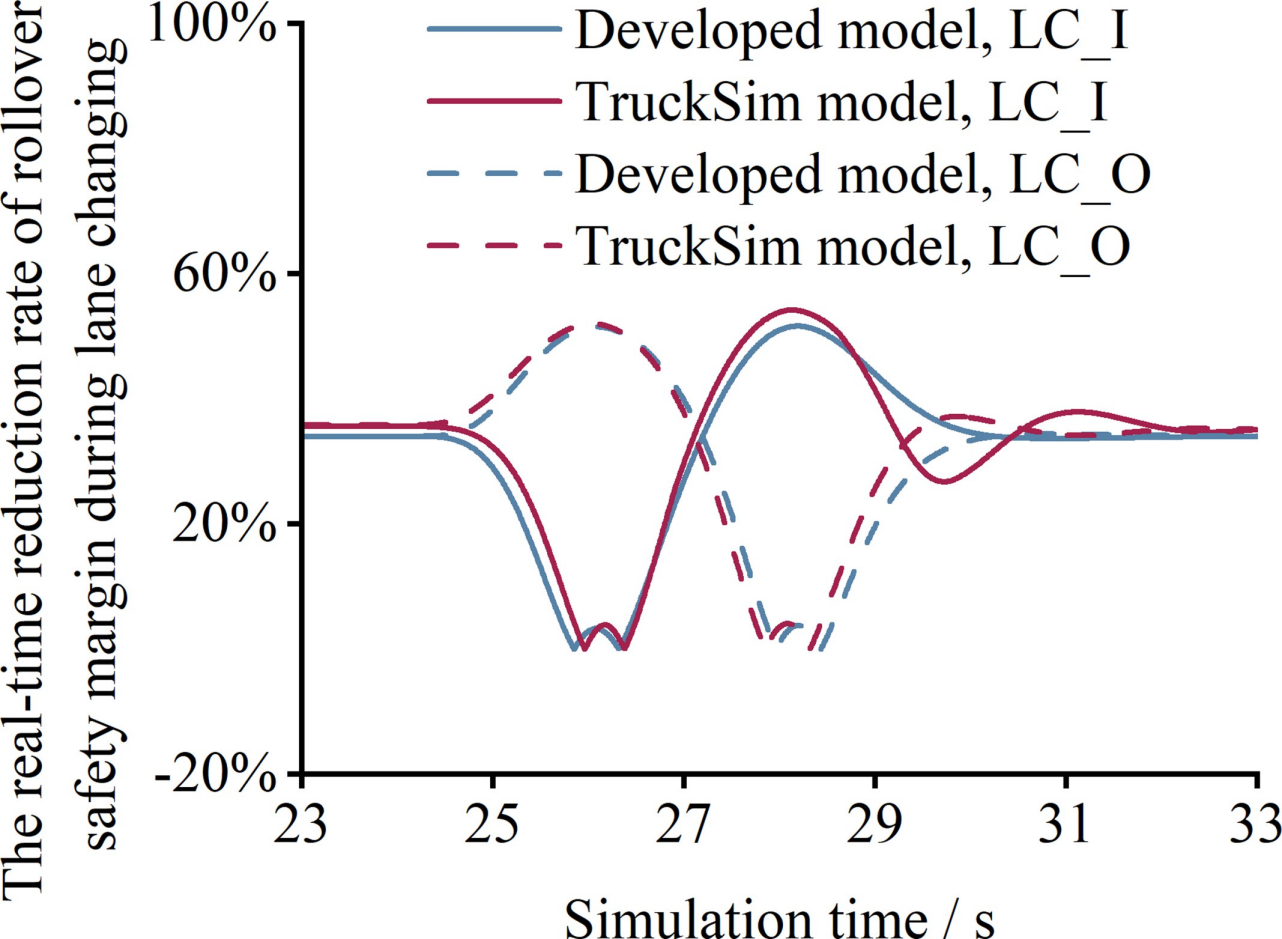
Comparison and validation of the results calculated by the TruckSim model and the developed model.

By applying the simulation process depicted in Figs 6 and 7, the real-time value of *f*_S_ is calculated for different lane-changing directions, as shown in Fig 8. The results indicate that both the TruckSim model and the developed model demonstrate good consistency, with the error in *f*_S_ not exceeding 8%. This finding reveals that the developed model can accurately represent the rollover safety margin of the tractor-semitrailers during lane changing. Furthermore, it can be employed for subsequent analyses to examine the effect of various lane-changing maneuvers and alignment characteristics on the vehicle’s rollover safety margin.

## 3. Results and discussion

This study aims to evaluate quantitatively the impact of emergency lane-change strategies on the rollover safety of tractor-semitrailers, employing the proposed indicator of rollover safety margin reduction rate. Firstly, the research analyzes the influence of the superelevation variable on the rollover safety margin, verifying the necessity of incorporating this variable into the dynamic model. Subsequently, the study analyzes the impact of curve radius, lane change speed, duration, and direction on the rollover safety margin. Lastly, the interactions between the vehicle and the road system and their effect on the rollover safety margin are assessed. Through this multifaceted analysis, insights are gleaned into how different emergency lane-change strategies and the geometric characteristics of horizontal curves influence the rollover safety margin for tractor-semitrailers. These insights contribute to the optimization of lane-change strategies.

### 3.1 The effect of superelevation and radius on the safety margin of rollover

**Fig 9:**
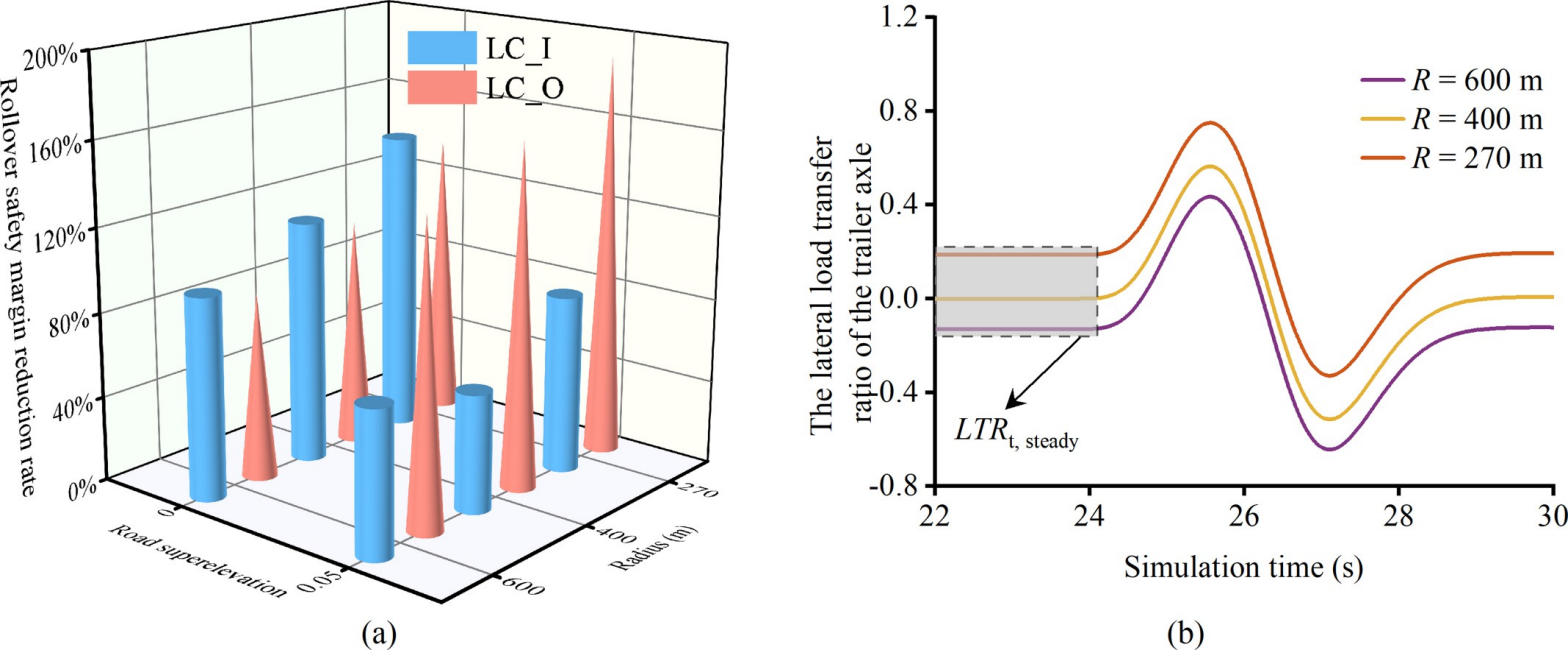
The effect of superelevation and radius on rollover safety margin of the tractor-semitrailer (*v*_*x*1_ = 80 km·h^-1^,*LCD* = 2 s). (a) *f*_S_. (b) The *LTR*_t_ during lane-changing maneuvers.

Section 2.1 introduces a critical hypothesis that the superelevation variable in horizontal curves plays a crucial role in analyzing the lateral stability of vehicles. This premise is supported by Fig 9(A), which illustrates the dependence of rollover safety on superelevation, contingent on the lane-change direction. Ignoring the superelevation variable in the dynamics model results in an overestimation of rollover safety for LC_I and an underestimation for LC_O, peaking at 59.9% and 54.6%, respectively, at the curve radius of 270 m. This phenomenon can be explained by Newton-Euler mechanics, where the superelevation-induced gravity component mitigates centrifugal force for LC_I, thus improving lateral stability. Conversely, superelevation exacerbates lateral load transfer for LC_O, diminishing stability. Xin et al. [52] supported these conclusions by estimating the critical safety speed for heavy truck overturning using the quasi-static rollover threshold, revealing that LC_O strategies reduce critical safety speeds. Therefore, vehicles employing LC_O are more likely to overturn at equivalent speeds. In conclusion, the superelevation variable represents a pivotal factor in dynamics modeling for tractor-semitrailers. A dynamic model that accounts for the superelevation variable can provide more precise results in assessing rollover safety, which is essential for secure lane-changing in vehicles, especially during hazardous situations.

In Fig 9(A), the effect of varying radii on the rollover safety margin of tractor-semitrailers is investigated. The results reveal that for LC_O, a decrease in curve radius is concomitant with a reduction in the rollover safety margin. This observation is consistent with classical mechanics principles, wherein a smaller curve radius leads to greater lateral deflection, resulting in an increased vehicle lateral load transfer rate and a higher *f*_S_ value. Alrejjal et al.’s study [53] highlighted the increased rollover susceptibility of loaded vehicles on sharp horizontal curves due to side winds but did not explore the impact of curve radius on the rollover safety margin, which is addressed in this paper. Interestingly, the trends in rollover safety margin for LC_I and LC_O are not identical, with the *f*_S_ being unexpectedly smaller for a 400 m radius than for a 600 m radius. This seeming contradiction to classical mechanics theory prompts further investigation. We focus on the *LTR* of the trailer axle (*LTR*_t_), analyzing its trend throughout the entire lane-change process. Utilizing *LTR* instead of *f*_S_ simplifies understanding and analysis, as *f*_S_ is a monotonic function of *LTR*, with consistent trends and one-to-one value correspondence (as shown in (17)). The analysis targets the trailer axle, as changes in *LTR* for the tractor-semitrailer’s steering, driving, and trailer axles are consistent during lane-changing, and the trailer axle’s *LTR*_t_ is invariably larger, making it the focal point of the analysis.

The trend of *LTR*_t_ during a lane change is illustrated in Fig 9(B). An anomalous change trend is found to be associated with the degree of superelevation compensating for the centrifugal force. For a curve radius of 270 m, *LTR*_t, steady_ during steady driving exceeds 0, indicating that the superelevation does not completely counterbalance the centrifugal force as the vehicle passes through the curve. Consequently, |*LTR*_t_|_max_ occurs at the crest of the wave during lane-changing. Maintaining the same lane change speed, an increase in the radius value causes the centrifugal force that has not been fully offset to diminish, and *LTR*_t, steady_ progressively converges to 0. |*LTR*_t_|_max_ during lane-changing correspondingly decreases (e.g., *R* = 400 m), and the smallest |*LTR*_t_|_max_ occurs when *LTR*_t, steady_ reaches 0. When the radius value exceeds 400 m, superelevation overcompensates the centrifugal force generated during the turn (e.g., *R* = 600 m). Consequently, *LTR*_t, steady_ becomes negative, and |*LTR*_t_|_max_ appears in the trough, exceeding the |*LTR*_t_|_max_ when *LTR*_t, steady_ is 0. Therefore, *f*_S_ increases with the curve radius in the local radius range where superelevation overcompensates the centrifugal force generated by the vehicle passing through the curve. This clarifies that the necessity for developing precise road radius and superelevation identification methods to formulate more dependable lane-changing control strategies. Although numerous studies have implemented various deep learning algorithms, amalgamating data from environmental perception devices like cameras, radar, and LiDAR for real-time lane marking and obstacle identification [54, 55], lightweight recognition of road superelevation continues to pose an unresolved challenge.

### 3.2 The effect of speed and duration of lane change on the safety margin of rollover

**Fig 10:**
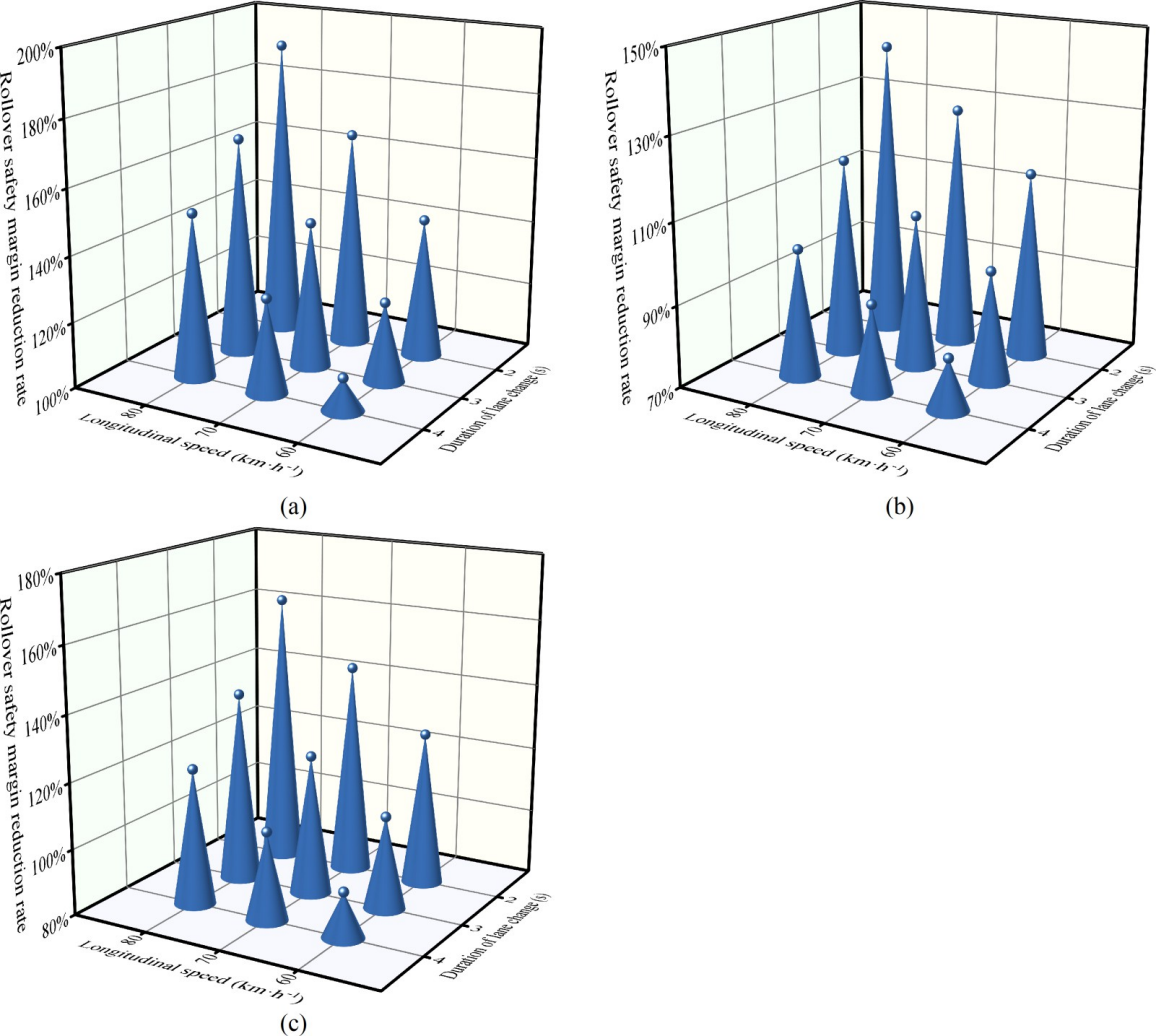
The effect of lane changing speed and duration on rollover safety margin (LC_O, *α*_h_ = 0.05). (a) *R* = 270 m. (b) *R* = 400 m. (c) *R* = 600 m.

Utilizing the rollover safety margin assessment method developed based on the improved 6-DOF model, the response characteristics of the rollover safety margin to variations in lane change speed and duration are analyzed. Fig 10 illustrates these characteristics for the tractor-semitrailer employing the lane change strategy of LC_O. The results demonstrate that the vehicle’s rollover safety margin is affected by the combined effects of lane change speed and duration. Specifically, the rollover safety margin decreases as speed increases and duration diminishes. For instance, the rollover safety margin for the tractor-semitrailer decreases by 91.5% at 70 km·h^-1^ (*LCD* = 4 s), 101.4% at 80 km·h^-1^ (with other variables constant), and 107.4% at a *LCD* of 3 s in a curve with a 600 m radius. It is critical to consider *f*_S_ values greater than 100%, as they indicate imminent rollover risk. From the perspective of marginal effect values (Tables 1 and 2), the marginal effect values of speed are increasing, while those of lane change duration are decreasing. This supports Kim’s findings [56] and suggests that high-speed emergency lane changes increase the likelihood of rollover collisions in tractor-semitrailers. In emergency situations, reducing speed and extending the duration of lane changes are critical to preventing vehicle rollovers. The method presented in this study offers a framework for determining both the deceleration magnitude and the extended duration of lane changes. While accurately controlling speed and lane-change duration is challenging for drivers due to their perception limitations, vehicles equipped with an active lane-changing function can readily achieve this. Most modern lane-changing algorithms focus on factors like longitudinal and lateral acceleration, smoothness, and fuel economy [57–59] with scant attention to rollover safety. This consideration is crucial for tractor-semitrailer emergency maneuvers due to their higher center of gravity, making them susceptible to rollovers [52]. The rollover safety margin reduction rate indicator introduced in this study can be incorporated into lane-changing algorithms as a constraint for cost functions. This measure captures both the vehicle-road dynamics and allows for the quantification of rollover safety margins.

**Table 1:** Marginal effect of speed of the tractor- semitrailer during lane change.

*R* / m	*v*_x1_ϵ[60, 70] / km·h^-1^	*v*_x1_ϵ[70, 80] / km·h^-1^
**270**	0.0190 / 0.0203 / 0.0232	0.0214 / 0.0224 / 0.0253
**400**	0.0131 / 0.0144 / 0.0174	0.0146 / 0.0156 / 0.0185
**600**	0.0090 / 0.0103 / 0.0132	0.0099 / 0.0108 / 0.0138

Note: The values **/**/** denote the marginal effect of lane changing speed at *LCD* = 4 s, 3 s and 2 s, respectively.

**Table 2:** Marginal effect of lane change duration of the tractor- semitrailer during lane change.

*R* / m	*LCD* ϵ [2, 3] / s	*LCD* ϵ [3, 4] / s
**270**	-0.0186 / -0.0216 / -0.0245	-0.0146 / -0.0159 / -0.0168
**400**	-0.0186 / -0.0216 / -0.0245	-0.0146 / -0.0159 / -0.0168
**600**	-0.0186 / -0.0216 / -0.0245	-0.0146 / -0.0159 / -0.0168

Note: The **/**/** signify the marginal effects of lane change duration under *v*_*x*1_ = 60 km·h^-1^, 70 km·h^-1^, and 80 km·h^-1^, respectively.

**Fig 11:**
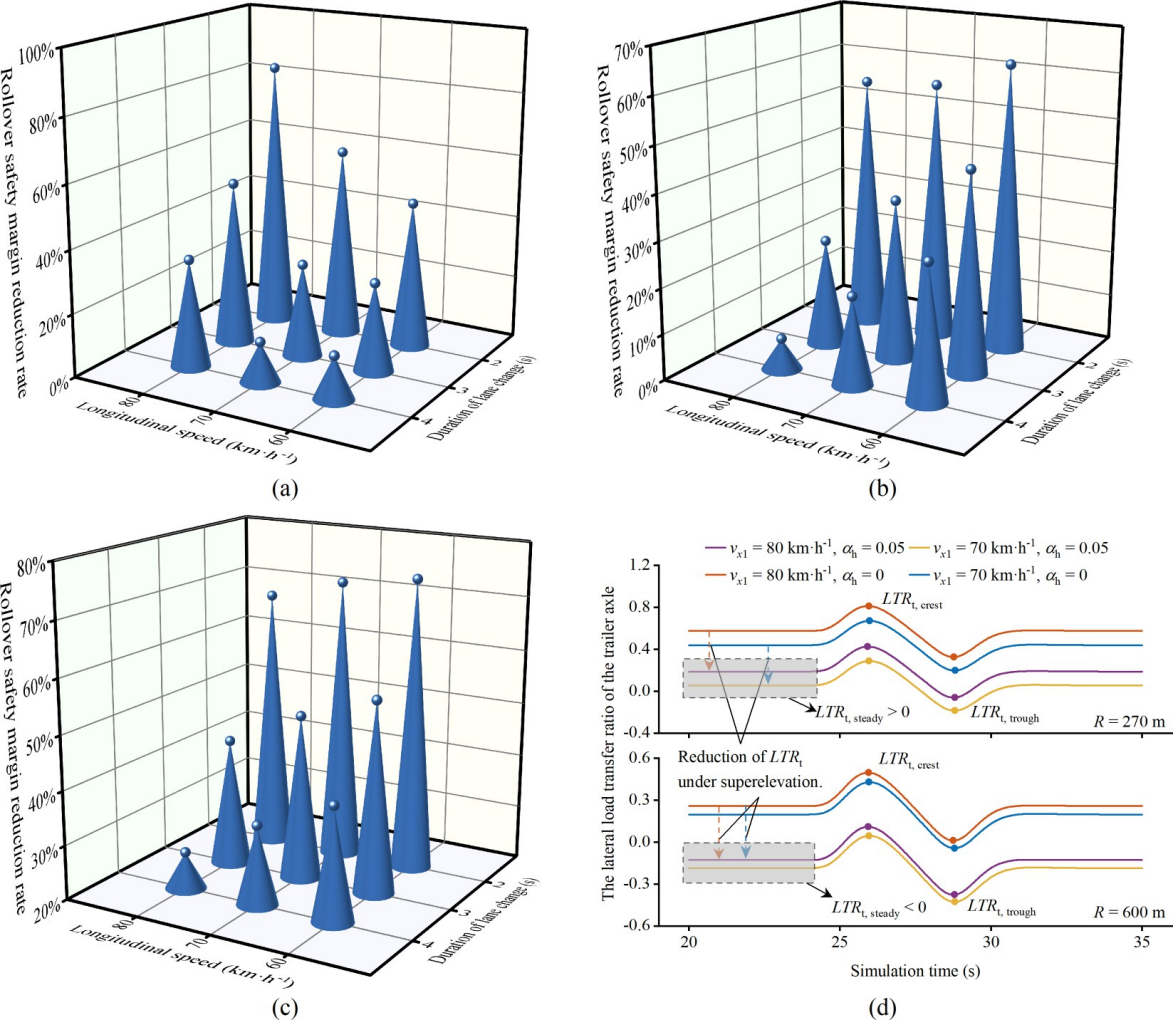
The *f*_S_ and *LTR*_3_ during lane change (LC_I, *α*_h_ = 0.05). (a) *R* = 270 m. (b) *R* = 400 m. (c) *R* = 600 m. (d) The *LTR*_t_ during lane-changing maneuvers (*LCD* = 4 s).

Fig 11 depicts the rollover safety margin response characteristics for the LC_I lane change direction. Notably, LC_I’s rollover safety response diverges from that of LC_O. For 400 m and 600 m radii, *f*_S_ exhibits a decline as speed increases. This trend is influenced by the degree to which superelevation counterbalances centrifugal force, analogous to the reason *f*_S_ increases with curve radius, as detailed in section 3.1. The *LTR*_t_ of the trailer axle remains the focal metric for analyzing the *LTR* trend during lane-changing, shown in Fig 11(D). For *α*_h_ = 0, *LTR*_t, steady_ for the vehicle in a steady state on the curve exceeds 0. Conversely, for *α*_h_ = 0.05, *LTR*_t, steady_ decreases, suggesting the countervailing effect of superelevation on the centrifugal force. Specifically, during lane changes at speeds of *v*_*x*1_ = 70 km·h^-1^ or 80 km·h^-1^ on a 270 m radius curve, the superelevation partially counteracts the vehicle’s centrifugal force on the curve. Consequently, in such conditions, *LTR*_t, steady_ is positive, with the |*LTR*_t_|_max_ during the lane change manifesting at the crest, labeled as *LTR*_t, crest_. While the negated centrifugal force due to superelevation remains consistent, elevated lane change speeds introduce a more significant centrifugal force, leading to a pronounced *LTR*_t, crest_. According to (17), *f*_S_ also increases.

However, for a curve radius of 600 m, the superelevation offsets the centrifugal force from the vehicle excessively. Consequently, the *LTR*_t, steady_ during steady driving is negative, and the |*LTR*_t_|_max_ during lane-changing emerges in the trough, referred to as *LTR*_t, trough_. With increasing lane change speed, both *LTR*_t, trough_ and *f*_S_ decline. The correlation between increased *f*_S_ and rising lane-changing speed can be attributed to the superelevation’s excessive compensation for the centrifugal forces generated by tractor-semitrailers. These observations suggest that reduced lane-changing speeds for tractor-semitrailers don’t always correspond to enhanced safety margins. A more effective strategy leverages the compensatory effects of superelevation against centrifugal force. Implementing this strategy necessitates accurate identification of road alignments and a thorough motion planning constraint set based on a vehicle dynamics model that factors in road characteristics. In autonomous vehicles, both offline and online Mapper subsystems compute maps of the driving environment, facilitating navigation in unstructured environments without collisions with static obstacles, such as road signs and curbs. The Motion Planner subsystem calculates the trajectory from the current state of the autonomous vehicle to the designated goal, closely approximating the path defined by the Behavior Selector subsystem while adhering to kinematic and dynamic constraints, thus ensuring passenger safety and comfort [60]. By integrating offline maps containing horizontal, longitudinal, and cross-sectional road parameter information, a vehicle dynamics model that considers geometric characteristics, and cost function constraints indexed by the rollover safety margin reduction rate into the aforementioned subsystems, and combining this with positional data from the vehicle navigation and positioning system, autonomous vehicles can proactively adapt speed planning to road conditions. This lightweight approach reduces the likelihood of rollover incidents during emergency lane-changing scenarios and will be the focus of our future research efforts.

### 3.3 The effect of lane change direction on the safety margin of rollover

**Fig 12:**
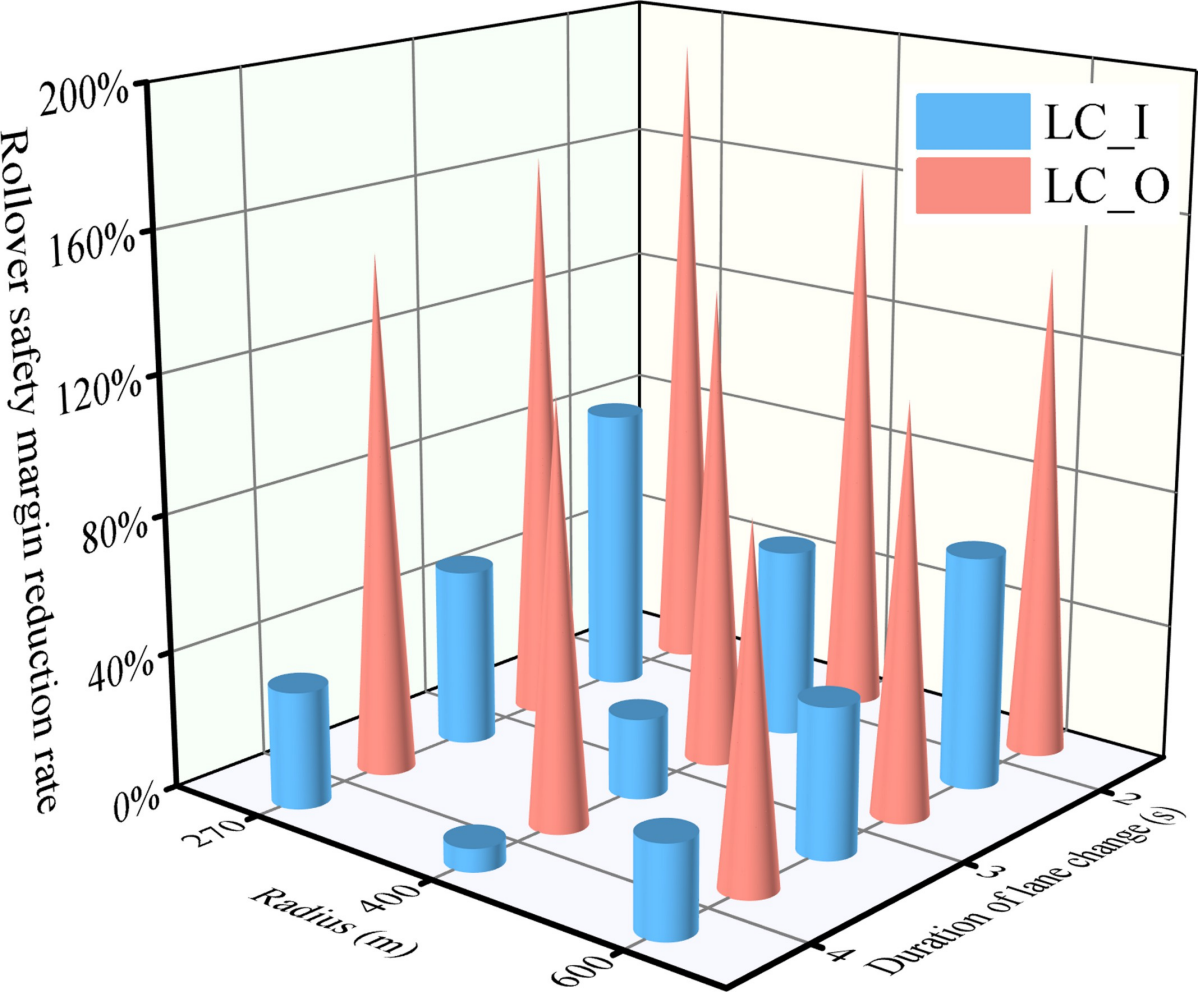
The effect of lane change direction on rollover safety margin of the tractor- semitrailer (*α*_h_ = 0.05, *v*_*x*1_ = 80 km·h^-1^).

The influence of superelevation on driving stability is impacted by the lane change direction [52], which subsequently affects the rollover safety of vehicles. However, the magnitude of this effect is not yet fully understood. Fig 12 illustrates the difference in rollover safety margin for the tractor-semitrailer under lane-changing directions LC_I and LC_O. With consistent road geometry, driving speed, and lane change time, LC_I exhibits a greater rollover safety margin compared to LC_O. Specifically, in the scenario presented in Fig 12 with *R* = 400 m and *LCD* = 2 s, the *f*_S_ of LC_I is 55.7%. In contrast, the the *f*_S_ of LC_O exceeds that of LC_I by 107.1%. This significant disparity underscores the safety advantage of selecting a lane change direction congruent with the curve bending direction; the opposite can lead to potential rollovers. Similar findings have been observed in a study on sport utility vehicles’ safety margins for sideslip and rollover, wherein a lane change direction inconsistent with the curve bending is linked to reduced rollover safety [61]. Thus, aligning the lane change direction with the curve’s bend is imperative for optimal vehicle stability, especially during emergency maneuvers.

### 3.4 The effect of interactions between the vehicle and road system on the safety margin of rollovers

The rollover safety margin of the tractor-semitrailer is determined by an interplay of several factors, including speed, lane change duration, lane change direction, and curve radius, as evidenced in Table 3. Adjustments to any combination of these variables can enhance the rollover safety margin. For example, while the emergency lane change in Case 1 may lead to a rollover, adjusting the direction in Case 4 or prolonging the lane change duration and concurrently reducing speed in Case 6 can prevent such occurrences. This finding aligns with the systems theory perspective which posits that the elements of road-vehicle system interplay, leading to accidents due to their dysfunctional interactions. No single element stands out as more critical than the others [7]. Consequently, a thorough assessment of the implications of lane change strategies informed by these factors is essential to mitigate rollover collision for tractor-semitrailers. During emergency lane changes on horizontal curves, it has been proven effective to align the lane change direction with the curvature direction of the curve. Additionally, extending the lane change duration and decelerating before the maneuver are advantageous. This insight is crucial for formulating lane-change trajectory planning and control algorithms, advocating for a direction that matches the curve’s bend, particularly during emergency situations.

**Table 3:** Coordinated effect of 4 independent variables on the safety margin of rollover.

Case	*v*_x1_ (km·h^-1^)	*LCD* (s)	Direction of lane change	*R* (m)	*f*_S_	Rollover
**1**	70	3	LC_O	400	122.7%	Yes
**2**	60	3	LC_O	400	108.3%	Yes
**3**	70	4	LC_O	400	106.8%	Yes
**4**	70	3	LC_I	400	35.2%	No
**5**	70	3	LC_O	600	107.4%	Yes
**6**	60	4	LC_O	400	93.7%	No
**7**	60	3	LC_O	600	97.1%	No
**8**	70	4	LC_O	600	91.5%	No

## 4. Conclusions

In scenarios requiring rapid responses, lane change maneuvers prove more effective than braking in averting rear-end collisions. However, these maneuvers produce significant lateral tipping torque, the magnitude of which is influenced by the road curvature. A paramount challenge lies in estimating the rollover safety margin during lane changes, especially considering the crucial interaction between the vehicle and the roadway. To navigate this intricacy, our research employs a tractor-semitrailer as the model, introducing a 6-DOF dynamic model incorporating a superelevation variable based on the traditional single-track model. Subsequently, a method is proposed to accurately quantify the vehicle-road interaction via the rollover safety margin reduction rate. This method is then validated using joint simulations in TruckSim^®^ and MATLAB/Simulink^®^. The study further investigates the impact of factors such as road superelevation, curve radius, lane change speed, duration, and direction on the rollover safety margin. It is demonstrated that the rollover safety margin reduction rate aptly characterizes the vehicle-road interaction during lane changes, facilitating the creation of safer lane change strategies. The primary conclusions derived are:

1. For tractor-semitrailers on horizontal curves, the direction of the emergency lane change most significantly influences the rollover safety margin, followed by the duration and speed of the change. Specifically, when analyzing the direction of the lane change, the safety margin reduction rate is discernibly lower when the vehicle changes lanes in the same direction as the curve, compared to changing lanes in the opposite direction. With respect to the duration of the lane change, extended lane change durations decrease the safety margin reduction rate, thus reducing rollover likelihood. Lastly, the speed at which the lane change occurs offers an intriguing observation. Owing to the varying compensatory effects of the curve’s superelevation on the centrifugal forces, the safety margin reduction rate for tractor-semitrailers doesn’t linearly increase with speed. Instead, it exhibits a "U"-shaped trend. This implies that lane changes at both extremely slow and extremely fast speeds can decrease the safety margin, whereas there exists an optimal speed range which minimizes this risk. This nuanced understanding can guide automated driving systems in making informed decisions to maximize safety during lane-changing maneuvers on horizontal curves.
2. The superelevation of the road has a dual effect on the emergency lane change maneuver on horizontal curves. If the change direction aligns with the curve, the superelevation effectively prevents vehicle rollover; however, in the opposite direction, it reduces the rollover safety margin. Simulation results indicate that the rollover safety margin’s reduction rate varies by over 40% based on the lane change direction. Therefore, incorporating superelevation into the classic single-track model, which characterizes the tractor-semitrailer’s dynamic lateral stability, is of paramount research importance. This model establishes a foundational framework for assessing rollover stability in autonomous vehicles more accurately.
3. The curve radius plays a pivotal role in influencing the rollover propensity of tractor-semitrailers. As radius decreases, the rollover safety margin for these vehicles diminishes. Consequently, emergency lane changes on tight-radius curves present substantial risks. Modifying the lane change’s duration, speed, or direction can mitigate rollover risks or avert potential collisions. While human drivers face challenges in accurately controlling speed and lane-change duration due to perceptual limitations, vehicles with active lane-changing capabilities can adeptly manage these tasks. Nonetheless, this proficiency is contingent upon the autonomous vehicle’s precise recognition of road geometry and a dynamics model incorporating road geometric characteristics. Notably, current road geometry recognition algorithms operate in real-time. This instantaneous recognition, while impressive, is not always imperative since road geometries tend to remain consistent over prolonged periods. Moreover, the existing algorithms tend to be intricate and have limited accuracy. The pursuit of lightweight and swift methods for accurate recognition of these parameters is still a challenging endeavor. Future research will concentrate on delivering optimized road geometric data, encompassing curve radius and superelevation.
4. The reduction rate of the rollover safety margin serves as a novel, open-ended index, reflecting the intricate system properties of vehicle-road interaction. To implement this in practice, one must first calibrate the rollover safety margin in the reference state using the improved 6-DOF dynamic model and then measure it in real-time during vehicle operation. The reference state is characterized as a situation where the vehicle travels steadily at its design speed on a minimum-radius curve, as stipulated by the current "Technical Standard of Highway Engineering" issued by China’s Ministry of Transport, without any lane changes or braking. Importantly, the index is relevant not only in evaluating the rollover safety margin of semi-trailers on horizontal curves but also in assessing other heavy vehicles. Its applicability transcends specific vehicle types since the rollover safety margin reduction rate is an indicator dependent on lateral load transfer—a characteristic not confined to particular vehicle categories. Accurate real-time estimation of lateral load transfer and precise discernment of road alignment parameters are crucial in this context. The present study, in constructing a 6-DOF dynamic model, considered factors such as road superelevation and curve radius to enhance the accuracy of lateral load transfer estimation. However, intricate scenarios necessitate further examination. For instance, weather-induced variations in road friction coefficient can substantially impact the vehicle-road interaction. Previous research has shown that elements like wet [62] or polished [63] pavements can diminish the road friction coefficient, consequently compromising vehicular rollover propensity [64]. Likewise, the sloshing of liquid-carrying articulated vehicles poses unique analytical challenges. Studies on liquid-carrying articulated vehicle stability have revealed that alterations in the vehicle’s center of gravity due to liquid sloshing can escalate rollover risk [65]. Future efforts will concentrate on overcoming these constraints and further validating the reduction rate index to broaden its applicability across various vehicle types and scenarios, with a specific focus on weather-related variations and the distinct dynamics of liquid-carrying articulated vehicles.

## Supporting information

S1 Table Nomenclature and nominal values used in the tractor-semitrailer model. (DOCX)

S1 File The coefficient matrices *P*_t_, *Q*_t_, *R*_t_, *C*_t_, and *D*_t_. (DOCX)
