# Supplementary material for: A rollover safety margin-based approach for quantifying the tractor-semitrailers’ emergency lane-changing response on expressway curves
Source: PLoS One. 2023 Sep 20;18(9):e0291783. doi: 10.1371/journal.pone.0291783 (PMC10511121; doi:10.1371/journal.pone.0291783)
Supplement: S1 Table — (DOCX) [file pone.0291783.s001.docx]

S1 Table

| **Symbol** | **Physical meaning** | **Nominal values / Unit** |
| --- | --- | --- |
| ***a*_1_** | longitudinal distance from the CG of the tractor to the front axle of the tractor. | 2.35 m |
| ***a*_2_** | longitudinal distance from the CG of the trailer to the hitch. | 5.61 m |
| ***B*_f_** | Axle track between the steering wheels of tractor. | 2.03 m |
| ***B*_r_** | Axle track between the driving wheels of tractor. | 1.86 m |
| ***b*_1_** | longitudinal distance from the CG of the tractor to the intermediate axle of the tractor. | 1.15 m |
| ***b*_2_** | longitudinal distance from the CG of the trailer to the front axle of the trailer. | 1.11 m |
| ***C*_f_** | Roll damping of the front suspension of the tractor. | 24,075 N⋅m⋅s⋅rad^-1^ |
| ***C*_r_** | Roll damping of the rear suspension of the tractor. | 24,075 N⋅m⋅s⋅rad^-1^ |
| ***C*_1_** | Roll damping of the front and rear suspensions of the tractor. | 48,150 N⋅m⋅s⋅rad^-1^ |
| ***C*_2_** | Roll damping of the trailer’s suspension. | 45,000 N⋅m⋅s⋅rad^-1^ |
| ***c*_1_** | longitudinal distance from the intermediate axle of the tractor to the hitch point. | 0.64 m |
| ***c*_2_** | longitudinal distance from the front axle of the trailer to the intermediate axle of the trailer. | 1.20 m |
| ***d*_1_** | longitudinal distance from the hitch point to the rear axle of the tractor. | 0.64 m |
| ***d*_2_** | longitudinal distance from the intermediate axle of the trailer to the rear axle of the trailer. | 1.20 m |
| ***F*_y1f_, *F*_y1m_, *F*_y1r_,** | Lateral force of the front, intermediate, and rear wheels of the tractor, respectively. | N |
| ***F*_y2f_, *F*_y2m_, *F*_y2r_,** | Lateral force of the front, intermediate, and rear wheels of the trailer, respectively. | N |
| ***F_x_*_h_, *F_y_*_h_, *F_z_*_h_** | Longitudinal, lateral and normal force of the trailer on tractor, respectively. | N |
| ***F*′*_x_*_h_, *F*′*_y_*_h_, *F*′*_z_*_h_** | Longitudinal, lateral and normal force of the tractor on trailer, respectively. | N |
| ***F*_zfo_, *F*_zfi_** | Normal force of the the inner and outer wheels of the tractor steering axle, respectively. | N |
| ***F*_zro_, *F*_zri_** | Normal force of the the inner and outer wheels of the tractor driving axle, respectively. | N |
| ***F*_zto_, *F*_zti_** | Normal force of the the inner and outer wheels of the trailer axle, respectively. | N |
| ***g*** | Gravitational acceleration. | 9.81 m⋅s^-2^ |
| ***h*_1s_** | Height of sprung mass CG of the tractor. | 1.18 m |
| ***h*_2s_** | Height of sprung mass CG of the trailer. | 2.19 m |
| ***h*_1r_** | Height of the roll center of the tractor sprung mass above the ground. | 0.61 m |
| ***h*_2r_** | Height of the roll center of the trailer sprung mass above the ground. | 1.02 m |
| ***h*_1cr_, *h*_2cr_** | Distances of the rolling center of the tractor and trailer sprung mass from the hitch point, respectively. | m |
| ***h*_1sr_, *h*_2sr_** | Distances of the rolling center of the tractor and trailer sprung mass from the CG, respectively. | m |
| ***h*_p_** | Height of hitch point. | 1.10 m |
| ***h*_fu_** | Distances of the rolling center of the tractor front suspension from the front axle. | m |
| ***h*_ru_** | Distances of the rolling center of the tractor rear suspension from the rear axle. | m |
| ***h*_tu_** | Distances of the rolling center of the trailer suspension from the trailer axle. | m |
| ***I*_1s_*_xx_*** | Mass moment of inertia of the tractor mass with respect to the *x*_1_ axis. | 2,283.9 kg⋅m^2^ |
| ***I*_2s_*_xx_*** | Mass moment of inertia of the trailer mass with respect to the *x*_2_ axis. | 21,802.3 kg⋅m^2^ |
| ***I*_1zz_** | Mass moment of inertia of the tractor mass with respect to the *z*_1_ axis. | 45,075.9 kg⋅m^2^ |
| ***I*_2zz_** | Mass moment of inertia of the trailer mass with respect to the *z*_2_ axis. | 285,516 kg⋅m^2^ |
| ***I*_1s_*_xz_*** | *x*_1_-*z*_1_ product of inertia of the tractor sprung mass. | 1,626 kg⋅m^2^ |
| ***I*_2sxz_** | *x*_2_-*z*_2_ product of inertia of the trailer sprung mass. | 0 kg⋅m^2^ |
| ***K*_x12_** | Roll viscous stiffness of saddle about x axis. | 5,729,578 N⋅m⋅rad^-1^ |
| ***K*_z12_** | Roll viscous stiffness of saddle about z axis. | 1,000,000 N⋅m⋅rad^-1^ |
| ***K*_f_** | Roll stiffness of the front suspension of the tractor. | 815,570 N⋅m⋅rad^-1^ |
| ***K*_r_** | Roll stiffness of the rear suspension of the tractor. | 815,570 N⋅m⋅rad^-1^ |
| ***K*_1_** | Roll stiffness of the front and rear suspensions of the tractor. | 1,631,140 N⋅m⋅rad^-1^ |
| ***K*_2_** | Roll stiffness of the trailer’s suspension. | 4265,880 N⋅m⋅rad^-1^ |
| ***k*_1f_** | Tire cornering stiffness of the front wheels of the tractor. | -231,430 N⋅rad^-1^ |
| ***k*_1m_** | Tire cornering stiffness of the intermediate wheels of the tractor. | -520,000 N⋅rad^-1^ |
| ***k*_1r_** | Tire cornering stiffness of the rear wheels of the tractor. | -520,000 N⋅rad^-1^ |
| ***k*_2f_** | Tire cornering stiffness of the front wheels of the trailer. | -553,000 N⋅rad^-1^ |
| ***k*_2m_** | Tire cornering stiffness of the intermediate wheels of the trailer. | -553,000 N⋅rad^-1^ |
| ***k*_2r_** | Tire cornering stiffness of the rear wheels of the trailer. | -553,000 N⋅rad^-1^ |
| ***M* _f_*_ξ_*, *M* _r_*_ξ_*** | Roll resistance moment of the front and rear of the tractor suspension, respectively. | N⋅m |
| ***M*_1_*_ξ_*, *M*_2_*_ξ_*** | Roll resistance moment of the tractor and trailer suspension, respectively. | N⋅m |
| ***m*_1_** | Mass of the tractor. | 6,360 kg |
| ***m*_2_** | Mass of the trailer. | 25,910 kg |
| ***m*_1s_** | Sprung mass of the tractor. | 4,455 kg |
| ***m*_2s_** | Sprung mass of the trailer. | 23,840 kg |
| ***m*_fu_, *m*_ru_, *m*_tu_** | Unsprung masses of the front, rear, and trailer, respectively. | kg |
| ***r*_1_** | Rolling radius of the wheels on the front axle of the tractor. | 0.52 m |
| ***r*_2_** | Rolling radius of the wheels on the rear axles of the tractor. | 0.52 m |
| ***r*_3_** | Rolling radius of the wheels on the trailer axles. | 0.52 m |
| ***ψ*_1_, *ψ*_2_** | Yaw rates of the tractor and trailer, respectivety. | rad⋅s^-1^ |
| ***v_x_*_1_, *v_x_*_2_** | Longitudinal speeds of the tractor and trailer, respectively. | m⋅s^-1^ |
| **ΔF_zf_, ΔF_zr_, ΔF_zt_** | Lateral load transfer of the front, rear, and trailer wheels, respectively. | N |
| ***α*_1f_, *α*_1m_, *α*_1r_** | Slip angles of the front, intermediate and rear wheels of the tractor, respectively. | rad |
| ***α*_2f_, *α*_2m_, *α*_2r_** | Slip angles of the front, intermediate and rear wheels of the trailer, respectively. | rad |
| ***α*_h_** | Superelevation. | m⋅m^-1^ |
| ***β*_1_, *β*_2_** | Sideslip angles of the tractor and trailer, respectivety. | rad |
| ***δ*_1f_** | Front wheel steering angle of the tractor. | rad |
| ***ξ*_1_, *ξ*_2_** | Roll angles of sprung mass of the tractor and trailer, respectively. | rad |
| ***γ*** | The angle between the tractor and trailer. | rad |
| ***k =* 1, 2**  **(Subscript)** | Tractor and trailer, respectivety. | - |
| ***i* = f, r, t**  **(Subscript)** | Front, rear, and trailer suspension, respectively. | - |
