# Supplementary material for: A rollover safety margin-based approach for quantifying the tractor-semitrailers’ emergency lane-changing response on expressway curves
Source: PLoS One. 2023 Sep 20;18(9):e0291783. doi: 10.1371/journal.pone.0291783 (PMC10511121; doi:10.1371/journal.pone.0291783)
Supplement: S1 File — (DOCX) [file pone.0291783.s002.docx]

S1 File

The coefficient matrices ***P***_t_, ***Q***_t_, ***R***_t_, ***C*_t_**, and ***D*_t_** in Eq (10) are given as follows:

$P_{\mathbf{t}}=\left[\begin{matrix} p_{11} & p_{12} & 0 & p_{14} & 0 & 0 & 0 & 0 \\ p_{21} & p_{22} & p_{23} & p_{24} & 0 & 0 & 0 & 0 \\ 0 & 0 & 1 & 0 & 0 & 0 & 0 & 0 \\ 0 & 0 & 0 & 0 & p_{45} & p_{46} & 0 & p_{48} \\ 0 & 0 & 0 & 0 & p_{55} & p_{56} & p_{57} & p_{58} \\ 0 & 0 & 0 & 0 & 0 & 0 & 1 & 0 \\ p_{71} & 0 & 0 & p_{74} & p_{75} & 0 & 0 & p_{78} \\ 1 & p_{82} & 0 & p_{84} & -1 & p_{86} & 0 & p_{88} \end{matrix}\right]$, $Q_{\mathbf{t}}=\left[\begin{matrix} q_{11} & q_{12} & 0 & 0 & 0 & 0 & 0 & 0 \\ q_{21} & q_{22} & q_{23} & 0 & 0 & 0 & q_{27} & 0 \\ 0 & 0 & 0 & 1 & 0 & 0 & 0 & 0 \\ 0 & 0 & 0 & 0 & q_{45} & q_{46} & 0 & 0 \\ 0 & 0 & q_{53} & 0 & q_{55} & q_{56} & q_{57} & 0 \\ 0 & 0 & 0 & 0 & 0 & 0 & 0 & 1 \\ q_{71} & q_{72} & 0 & 0 & q_{75} & q_{76} & 0 & 0 \\ 0 & -1 & 0 & 0 & 0 & 1 & 0 & 0 \end{matrix}\right]$

$$R_{\mathbf{t}}=\left[\begin{matrix} -\left(a_{1}+b_{1}+c_{1}\right)k_{1\text{f}} & -h_{1\text{cr}}k_{1\text{f}} & 0 & 0 & 0 & 0 & -k_{1\text{f}} & 0 \\ m_{1}\left(b_{1}+c_{1}\right)g & -2m_{1\text{s}}h_{1\text{sr}}g+m_{1}gh_{1cr} & 0 & m_{2}a_{2}g & -2m_{s2}h_{\text{s}2}g+m_{2}gh_{2cr} & 0 & \left(m_{1}+m_{2}\right)g & 0 \end{matrix}\right]^{\text{T}}$$

***C*_t_** = ***E***_8×8_，***E*** is unit matrix,

***D*_t_** = ***O*** _8×2_，***O*** is zero matrix.

The relevant elements in the constant matrix ***P*_t_** are as follows:

$p_{11}=m_{1}v_{x1}\left(b_{1}+c_{1}\right)$, $p_{12}=I_{1zz}$, $p_{14}=-\left(m_{1s}h_{1sr}\left(b_{1}+c_{1}\right)+I_{1sxz}\right)$,

$p_{21}=m_{1}v_{x1}h_{1cr}-m_{1s}h_{1sr}v_{x1}$, $p_{22}=-I_{2zz}$, $p_{23}=C_{1}^{*}$, $p_{24}=-m_{1s}h_{1sr}h_{1cr}+I_{1sxx}+2m_{1s}h_{1sr}^{2}$,

$p_{45}=m_{2}v_{x2}a_{2}$, $p_{46}=-I_{2zz}$, $p_{48}=-m_{2s}h_{2sr}a_{2}+I_{2sxz}$,

$p_{55}=m_{2}v_{x2}h_{2cr}-m_{2s}h_{2sr}v_{x2}$, $p_{56}=-I_{2sxz}$, $p_{57}=C_{2}^{*}$, $p_{58}=-m_{2s}h_{2sr}h_{2cr}+I_{2sxx}+2m_{2s}h_{2sr}^{2}$,

$p_{71}=m_{1}v_{x1}$, $p_{74}=-m_{1s}h_{1sr}$, $p_{75}=m_{2}v_{x2}$, $p_{78}=-m_{2s}h_{2sr}$,

$p_{82}=\frac{-\left(b_{1}+c_{1}\right)}{v_{x1}}$, $p_{84}=-\frac{h_{1cr}}{v_{x1}}$, $p_{86}=-\frac{a_{2}}{v_{x2}}$, $p_{88}=\frac{h_{2cr}}{v_{x2}}$.

The relevant elements in the constant matrix ***Q*_t_** are as follows:

$q_{11}=\left(a_{1}+b_{1}+c_{1}\right)k_{1f}+c_{1}k_{1m}-d_{1}k_{1r}$,

$q_{12}=\frac{a_{1}\left(a_{1}+b_{1}+c_{1}\right)k_{1f}-b_{1}c_{1}k_{1m}+d_{1}\left(b_{1}+c_{1}+d_{1}\right)k_{1r}}{v_{x1}}-m_{1}v_{x1}\left(b_{1}+c_{1}\right)$,

$q_{21}=h_{1cr}\left(k_{1f}+k_{1m}+k_{1r}\right)$,

$q_{22}=\frac{\left(a_{1}k_{1f}-b_{1}k_{1m}-\left(b_{1}+c_{1}+d_{1}\right)k_{1r}\right)h_{1cr}}{v_{x1}}-m_{1}v_{x1}h_{1cr}+m_{1s}v_{x1}h_{1sr}$,

$q_{23}=m_{1s}gh_{1sr}-K_{1}-K_{x12}$, $a_{27}=K_{x12}$,

$q_{45}=\left(a_{2}+b_{2}\right)k_{2f}+\left(a_{2}+b_{2}+c_{2}\right)k_{2m}+\left(a_{2}+b_{2}+c_{2}+d_{2}\right)k_{2r}$,

$q_{46}=\frac{\begin{matrix} -b_{2}\left(a_{2}+b_{2}\right)k_{2f}-\left(b_{2}+c_{2}\right)\left(a_{2}+b_{2}+c_{2}\right)k_{2m} \\ -\left(b_{2}+c_{2}+d_{2}\right)\left(a_{2}+b_{2}+c_{2}+d_{2}\right)k_{2r} \end{matrix}}{v_{x2}}-m_{2}v_{2x}a_{2}$,

$q_{53}=K_{x12}$, $q_{55}=h_{2cr}\left(k_{2f}+k_{2m}+k_{2r}\right)$,

$q_{56}=\frac{\left(-b_{2}k_{2f}-\left(b_{2}+c_{2}\right)k_{2m}-\left(b_{2}+c_{2}+d_{2}\right)k_{2r}\right)h_{2cr}}{v_{x2}}-m_{2}v_{2x}h_{2cr}+m_{2s}v_{2x}h_{2sr}$, $q_{57}=m_{2s}gh_{2sr}-K_{1}-K_{x12}$,

$q_{71}=k_{1f}+k_{1m}+k_{1r}$, $q_{72}=\frac{a_{1}k_{1f}-b_{1}k_{1m}-\left(b_{1}+c_{1}+d_{1}\right)k_{1r}}{v_{x1}}-m_{1}v_{x1}$, $q_{75}=k_{2f}+k_{2m}+k_{2r}$,

$q_{76}=\frac{-b_{2}k_{2f}-\left(b_{2}+c_{2}\right)k_{2m}-\left(b_{2}+c_{2}+d_{2}\right)k_{2r}}{v_{x2}}-m_{2}v_{x2}$.
